# Detection of pup odors by non-canonical adult vomeronasal neurons expressing an odorant receptor gene is influenced by sex and parenting status

**DOI:** 10.1186/s12915-016-0234-9

**Published:** 2016-02-15

**Authors:** Thiago S. Nakahara, Leonardo M. Cardozo, Ximena Ibarra-Soria, Andrew D. Bard, Vinicius M. A. Carvalho, Guilherme Z. Trintinalia, Darren W. Logan, Fabio Papes

**Affiliations:** Department of Genetics and Evolution, Institute of Biology, University of Campinas, Rua Monteiro Lobato, Campinas, SP 13083-862 Brazil; Graduate Program in Genetics and Molecular Biology, Institute of Biology, University of Campinas, Campinas, SP 13083-862 Brazil; Wellcome Trust Sanger Institute, Wellcome Genome Campus, Hinxton, Cambridge CB10 1SA UK; Monell Chemical Senses Center, 3500 Market St., Philadelphia, PA 19104 USA; Current affiliation: Neurosciences Graduate Program, University of California San Diego, 9500 Gilman Drive 0634, La Jolla, CA 92093-0634 USA; Current affiliation: MRC Centre for Developmental Neurobiology, King’s College London, Strand, London, WC2R 2LS UK

**Keywords:** Olfaction, Odorant receptor, Pup odors, Sexual dimorphism, Social behavior, Vomeronasal organ

## Abstract

**Background:**

Olfaction is a fundamental sense through which most animals perceive the external world. The olfactory system detects odors via specialized sensory organs such as the main olfactory epithelium and the vomeronasal organ. Sensory neurons in these organs use G-protein coupled receptors to detect chemosensory stimuli. The odorant receptor (OR) family is expressed in sensory neurons of the main olfactory epithelium, while the adult vomeronasal organ is thought to express other types of receptors.

**Results:**

Here, we describe *Olfr692*, a member of the *OR* gene family identified by next-generation RNA sequencing, which is highly upregulated and non-canonically expressed in the vomeronasal organ. We show that neurons expressing this gene are activated by odors emanating from pups. Surprisingly, activity in *Olfr692*-positive cells is sexually dimorphic, being very low in females. Our results also show that juvenile odors activate a large number of *Olfr692* vomeronasal neurons in virgin males, which is correlated with the display of infanticide behavior. . In contrast, activity substantially decreases in parenting males (fathers), where infanticidal aggressive behavior is not frequently observed.

**Conclusions:**

Our results describe, for the first time, a sensory neural population with a specific molecular identity involved in the detection of pup odors. Moreover, it is one of the first reports of a group of sensory neurons the activity of which is sexually dimorphic and depends on social status. Our data suggest that the *Olfr692* population is involved in mediating pup-oriented behaviors in mice.

**Electronic supplementary material:**

The online version of this article (doi:10.1186/s12915-016-0234-9) contains supplementary material, which is available to authorized users.

## Background

One of the fundamental properties of the nervous system in all animal species is its ability to detect and interpret sensory information. Most mammals use olfaction to detect the presence and quality of food, predators, competitors, and potential mates. The olfactory system of mammals evolved several subsystems in the nasal cavity, each with its own sensory organ, molecular receptors, and pathways in the brain [1]. The main olfactory epithelium (MOE), regarded as the site of detection for volatile odorants, harbors olfactory sensory neurons (OSNs). Each OSN canonically expresses one gene in the large odorant receptor (*OR*) gene family [2–5].

Besides the MOE, detection of olfactory stimuli is also accomplished by a second sensory structure in the nose, the vomeronasal organ (VNO) [1, 6]. The VNO has been extensively implicated in the mediation of a range of instinctive responses triggered by intra- and interspecies olfactory cues [7], such as male-male aggression [8], mating and gender discrimination [9–11], the inhibition of juvenile-oriented sexual behavior [12], female lordosis sexual behavior [13], and defensive behavior towards predators [14]. VNO sensory neurons (VSNs) express receptors in the V1R [15] or V2R [16–18] families of vomeronasal receptors (VRs) and in the formyl-peptide receptor family [19].

Here, we used a combination of next-generation sequencing, molecular biology, and histochemical analyses to show that a gene coding for a receptor in the OR family is highly expressed in a defined and non-canonical subpopulation of adult VNO cells, characterized by the expression of a unique set of molecular markers. We also show that cells expressing this receptor gene, though not responsive to most known intra- and interspecies VNO stimuli, are activated by scents from pups. Moreover, this subpopulation is robustly activated in virgin males, whereas activation is low in fathers, virgin females, and mothers. These results indicate that activity in such pup odor-responsive VNO neurons is sexually dimorphic and depends on the animal’s social status, suggesting that they may mediate pup-oriented behaviors in adult mice.

## Results

### Deep sequencing and quantitative PCR reveal high level expression of *Olfr692*, an odorant receptor family gene, in the adult VNO

A subset of VSNs from adult mice has been shown to detect volatile odorants [20], ligands usually associated with OSNs and OR receptors in the MOE [7]. Thus, it is conceivable that some VNO cells may express OR receptors. To investigate the expression of ORs in the adult VNO, we mined Illumina next-generation deep RNA sequencing data from adult VNO samples (initial description and validation of these libraries can be found in [21]).

**Fig. 1:**
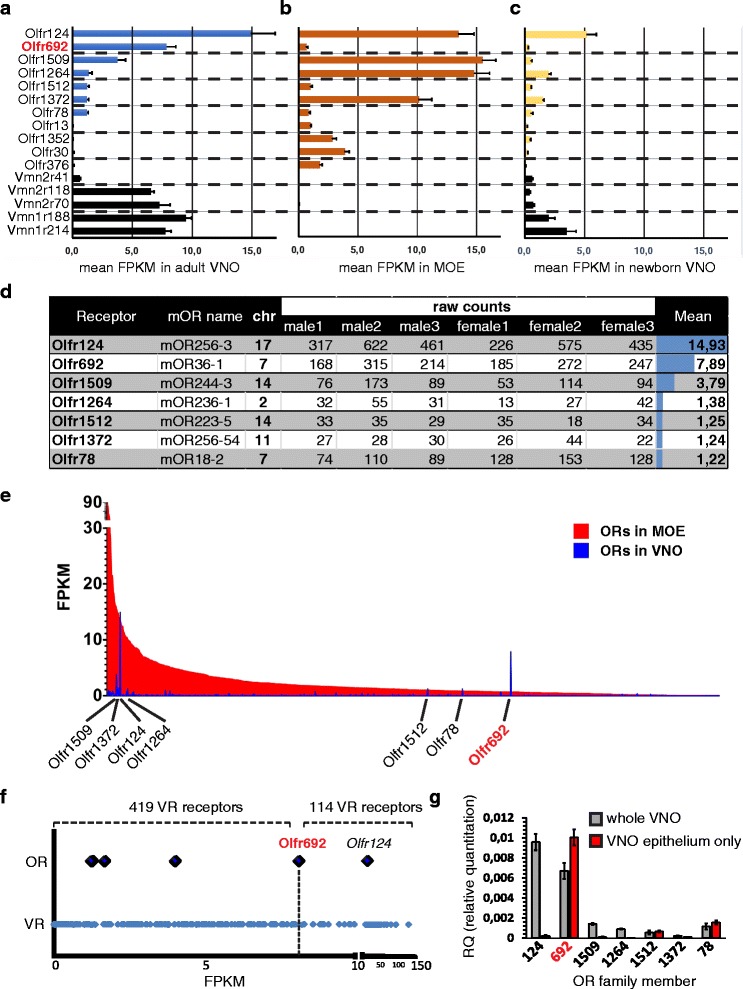
Next generation RNA sequencing and quantitative PCR reveal high and unique expression of an odorant receptor (*OR*) gene in the mouse vomeronasal organ. (**a**–**c**) RNA sequencing expression data for selected genes represented as mean fragments per kilobase of annotated sequence per million reads (FPKM). Error bars are the standard error of the mean (SEM). The first seven *OR* genes have FPKM >1.0 in the vomeronasal organ (VNO) library; the remaining *OR* genes have been reported to be expressed in juveniles [23]. Black bars represent vomeronasal receptor (*VR*) genes, for comparison. Expression of *Olfr692*, marked in red, stands out as high in the VNO, higher in the VNO than in the main olfactory epithelium (MOE), and comparable to the expression of *VR* genes. (**a**) Adult VNO libraries (n = 6 libraries; each library was from VNOs pooled from three individuals); (**b**) Adult MOE libraries (n = 6 libraries; each library was from one individual); (**c**) Juvenile libraries (n = 3 libraries; each library was from VNOs pooled from three to four individuals). (**d**) Seven *OR* genes with mean FPKM values >1.0 in adult VNO libraries, showing no difference in expression between males and females. 'mOR name' refers to alternate mOR nomenclature [2]. Raw counts are number of reads uniquely mapped to genomic model. The right column indicates mean FPKM in the VNO. Chr, mouse genome chromosomal location. *OR* genes with FPKM values >1.0 are located in distinct chromosomes, suggesting that their expression in the VNO is not related to their genomic position. (**e**) Comparison between FPKM values for *OR* genes in the adult MOE (red bars) and VNO (blue bars), illustrating that *Olfr692* expression is uniquely VNO-enriched. The x-axis is ordered according to expression level in the MOE. (**f**) Comparison between FPKM for *OR* (large diamonds) and *VR* (small diamonds) genes in the VNO. The two most abundant *OR* genes, *Olfr124* and *Olfr692*, are labeled. The top part shows the number of *VR* genes with FPKM values lower or higher than *Olfr692* (419 *VR* genes, including 171 pseudogenes, exhibit lower values; 114 *VR* genes, including five pseudogenes, exhibit higher values). The x-axis scale is different to the left and right of an interruption at FPKM = 10. (**g**) Real-time qPCR using TaqMan probes to validate the expression of *OR* genes which have mean FPKM values >1.0 in the adult VNO RNA seq libraries. The y-axis indicates relative expression level (RQ) as compared to the expression of β-actin. Gray bars indicate expression levels in whole VNO samples and red bars represent the finely dissected VNO neuroepithelium. Error bars are SEM (n = 4 animals, three technical replicates)

We found that seven *OR* genes had mean fragments per kilobase of exon sequence per million fragments (FPKM) values higher than 1.0 in the VNO (Fig. 1a). Interestingly, only one of these genes, *Olfr692*, stands out as being expressed at much higher levels in the VNO (Fig. 1a) than in the MOE (Fig. 1b,e), where *OR* genes are canonically expressed. The remaining six *OR* genes are either expressed at equivalent levels in the MOE and VNO or have much higher expression in the MOE (Fig. 1a,b,e).

Moreover, the expression of *Olfr692* is singularly high in the VNO RNA sequencing libraries, with FPKM values comparable to canonically expressed VR genes in the V1R and V2R families, such as *Vmn2r118* (V2R family) and *Vmn1r188* (V1R family) (Fig. 1a). *Olfr692* expression stands out as being higher than the expression of 419 *VR*s, with only 114 *VR*s expressed more abundantly (Fig. 1f). Moreover, the expression of *Olfr692* is 13 times higher than the median *VR* expression and 1.3 times greater than the mean *VR* expression. Together, these data suggest that the expression of *Olfr692* is significantly high and appreciable in the VNO.

The expression of two other *OR* genes, *Olfr124* and *Olfr1509*, is also high in the VNO libraries (Fig. 1a,e), but these genes are known to be expressed in another chemosensory structure in the nasal cavity, the Septal Organ of Masera (SOM), at very high levels (>50 % and >12 % of SOM cells, respectively) [22]. Because the VNO libraries we used for RNA sequencing were made from whole VNO preparations, including not only the sensory epithelium, but also progenitor and non-neural supporting cells, underlying glandular tissue, blood vessels, and the lower part of the nasal septum [21], it is possible that a limited fraction of SOM cells may have been included in the RNA used to construct our VNO libraries. This may account for the high FPKM values of some *OR* genes in the VNO, including *Olfr124* and *Olfr1509*.

Therefore, we performed real-time qPCR to validate the expression of *OR* genes found in the RNA seq VNO libraries and to assess if they are expressed in the vomeronasal neuroepithelium (Fig. 1g). In this experiment, we analyzed the expression of each of the seven *OR* genes, comparing a whole VNO prep sample versus a finely dissected VNO epithelium sample. We exercised extra caution during fine dissection of the VNO to avoid contamination with SOM cells in the nasal septum. When a whole VNO sample was under analysis, all seven most highly expressed *OR* genes in the RNA seq libraries had qPCR expression levels that matched their expression abundance in the RNA sequencing experiments (Fig. 1g). In contrast, when we analyzed the expression of such genes in the finely dissected VNO sample, only *Olfr692* had a significantly high relative abundance in the VNO epithelium (Fig. 1g). The expression of the remaining *OR* genes, *Olfr124*, *Olfr1509*, *Olfr1264,Olfr1512*, *Olfr78*, and the pseudogene *Olfr1372*, was absent or very low in the VNO epithelium (Fig. 1g).

Together, our RNA sequencing and qPCR data suggest that a few *OR* genes, notably *Olfr692*, are expressed in the adult VNO neuroepithelium.

### *Olfr692* is highly and uniquely expressed in the adult VNO

Out of the seven *OR* genes expressed in the VNO RNA seq libraries, only *Olfr78*, which is expressed at much lower levels than *Olfr692* according to the qPCR data (Fig. 1g), has been previously investigated [23]. Its expression, along with that of a few other *OR* genes, was reported to be almost absent in the adult VNO, being virtually restricted to the young, where a small subset of *OR* genes is expressed by few VNO cells [23]. Therefore, we decided to investigate whether the *OR* genes identified in our adult VNO RNA sequencing libraries are also expressed in newborn mice. We dissected whole VNOs from P0.5 animals and pooled three to four individuals to construct libraries for RNA sequencing. Most receptors are expressed at reduced levels in VNOs from P0.5 animals (Fig. 1c). Nonetheless, the expression levels across the whole newborn receptor repertoire are correlated with the expression levels in the adult (rho = 0.67, *P* <2.2 × 10^-16^), suggesting that the distribution observed in adults has already started to be shaped at an early stage. In newborns, we observed expression of the seven ORs previously identified in adults (Fig. 1c).

Notably, however, the *Olfr692* gene stands out again due to its significantly lower expression in newborns as compared to adults (Fig. 1a,c): for most *OR* genes, the expression in the adult VNO libraries is up to three times higher than the expression in newborns, but *Olfr692* is exceptional in that its FPKM value in the adult is 26 times greater than in the newborn RNA seq library (Fig. 1a,c). These data show that the *Olfr692* gene is differentially expressed in the adult VNO and suggest that this differential expression is much more prominent for this gene than for all other olfactory receptor genes expressed in the vomeronasal system.

### *Olfr692* is uniquely expressed in a defined subpopulation of adult VNO neurons

To investigate the spatial localization of cells expressing *OR* genes and to confirm which of them are expressed in the VNO sensory epithelium, we performed chromogenic and fluorescent in situ hybridization (ISH) experiments on cryostat VNO sections, using probes specifically designed to discriminate with great accuracy among the closely related genes in the *OR* family.

**Fig. 2:**
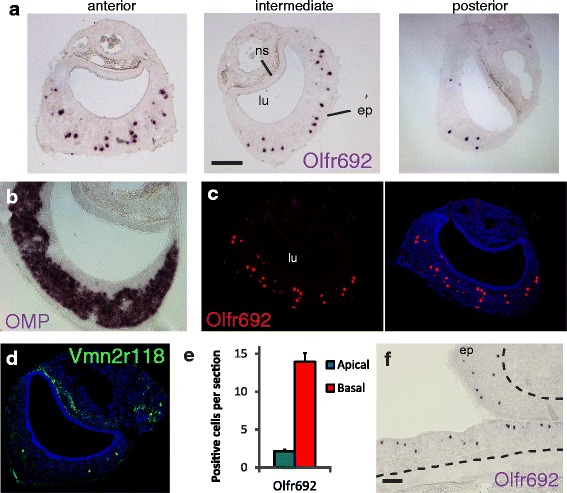
The *Olfr692* OR gene is expressed in a large subpopulation of cells in the adult mouse vomeronasal organ. (**a**) In situ hybridization (ISH) on 16-μm cryostat VNO sections with a cRNA DIG-labeled probe for *Olfr692*. Chromogenic development stains a defined subpopulation of sparsely distributed cells (purple), along the anterior-posterior VNO axis, with no apparent spatial segregation (representative images from a set 48 sections, from 20 mice). (**b**) Chromogenic ISH on a VNO section with DIG-labeled probe for Olfactory Marker Protein (*OMP*), coding for OMP, expressed in all VSNs. (**c**) Fluorescent ISH on VNO sections with DIG-labeled probe for *Olfr692* (red fluorescence; representative images from a set of 20 sections, from 20 individuals). (**d**) Fluorescent ISH with DNP-labeled probe for *Vmn2r118* (green). See Additional file 1: Figure S1b–d for other V2R receptors. (**e**) Quantitation of *Olfr692*-positive cells in apical (blue bars) or basal (red bars) zones of the VNO (error bars are SEM; n = 41 subjects; two to three sections per subject). (**f**) Chromogenic ISH on an MOE section with DIG-labeled *Olfr692* probe (representative images from a set of nine sections, from three individuals). ep, sensory epithelium; lu, vomeronasal organ lumen; ns, non-sensory tissue and blood vessels. Scale bars represent 100 μm. Blue labeling indicates To-Pro-3 nuclear staining. See also results with control *OR* probes in Additional file 1: Figure S1a

*Olfr692*, the *OR* gene with the highest expression in the VNO according to the real-time PCR experiments (Fig. 1g), had its expression consistently confirmed in the VNO by ISH: we found *Olfr692*-positive staining in a defined subpopulation of cells (Fig. 2a,c) sparsely distributed within the neuroepithelium in sections across the anterior-posterior axis (the epithelium is evidenced by the expression of Olfactory Marker Protein gene, *Omp*, in Fig. 2b).

In VNOs from adult mice above the age of 3 months, we found 16.2 ± 0.5 *Olfr692*-positive cells per histological section (mean ± SEM; n = 48 sections, four sections per mouse; Fig. 2a). Moreover, the number of cells expressing *Olfr692* is equivalent to or higher than that of canonical VRs. For example, when we used a probe that specifically recognizes *Vmn2r118*, a member of the V2R *VR* gene family, we found 5.5 ± 0.5 *Vmn2r118*-positive cells per section (mean ± SEM; n = 12 sections, two sections per mouse; Fig. 2d). Similar results were obtained with probes based on other *VR* genes (Additional file 1: Figure S1b–d; Additional file 2: Dataset S1). Together, these data show that the *Olfr692* gene in the *OR* family, though non-canonically expressed in the VNO, is robustly expressed in this sensory organ, an expression on average comparable to or higher than the expression of its *VR* genes.

In terms of spatial distribution, *Olfr692*-positive cells are concentrated in the basal layer of the VNO (Fig. 2e), which is characterized by the expression of V2R family receptors and the associated Gαo subunit of heterotrimeric G protein [1]. Though sparsely distributed across the epithelium (Fig. 2a), *Olfr692*-positive VNO cells are more densely packed than in the MOE, where this *OR* gene is expressed in the epithelium’s zone II (Fig. 2f; MOE zones were defined according to [4]).

In contrast, *Olfr78*, the gene with the second highest expression level in our qPCR analysis (Fig. 1g), is expressed in only 3.3 ± 0.4 VNO cells per section (mean ± SEM; n = 12 sections, two sections per mouse; Additional file 1: Figure S1e), a much more limited pattern of expression in comparison with *Olfr692* (Fig. 2a). This is in keeping with the low reported expression of this *OR* gene in the adult VNO [23]. Moreover, *Olfr78* expression is concentrated in the apical portion of the chemosensory epithelium (Additional file 1: Figure S1f), in agreement with its previously described co-expression with the G protein Gαi2 subunit, a known marker of the VNO apical region [23]. Expression of *Olfr1512* is also limited to a few cells per section (Additional file 1: Figure S1g), consistent with its low expression level in both RNA sequencing and qPCR experiments (Fig. 1a,g). Expression of *Olfr124* and *Olfr1509*, *OR* genes with the first and third highest mean FPKM values in our VNO libraries but no detectable expression in the purified vomeronasal epithelium by qPCR (Fig. 1a,g), was not found in the vomeronasal epithelium by ISH (Additional file 1: Figure S1h, i), even though the same probes detect an extensive amount of cells in the MOE (Additional file 1: Figure S1h,j). In agreement with previous reports [22], expression of *Olfr124* and *Olfr1509* is robust in the SOM (Additional file 1: Figure S1k; see also [22]). Together, these data indicate that the expression of both *OR* genes is absent in the VNO and that their presence in the VNO RNA sequencing libraries was probably due to a small number of SOM cells included in the whole VNO RNA preparations.

Our transcriptomic and histological results show that just one out of all *OR* genes, *Olfr692*, is expressed in a large number of chemosensory cells in the sensory epithelium of the adult mouse VNO, a robust expression comparable to that of other *VR* genes at the same age.

### Temporal pattern of *Olfr692* expression in the mouse VNO

**Fig. 3:**
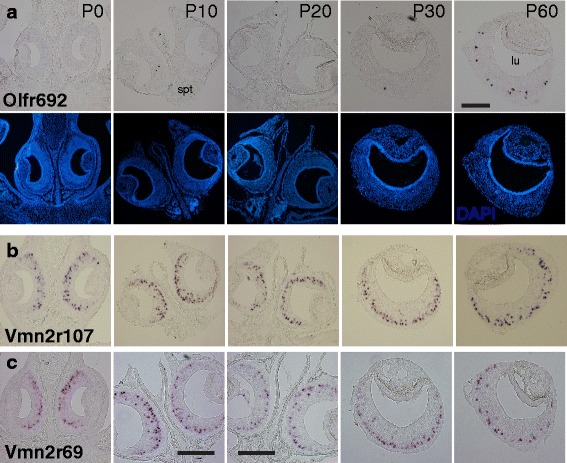
*Olfr692* is expressed in a developmentally regulated manner, with maximum expression in adults older than 2 months. (**a**) Chromogenic in situ hybridization to investigate the temporal pattern of *Olfr692* expression in the VNO during postnatal development (P0 to P60). At each age, images are representative from a set of 32 sections, from four individuals. (**b**, **c**) Temporal expression of V2R receptors in subclade A8 (**b**), investigated with probe for receptor gene *Vmn2r107*, and subclade A5 (**c**), investigated with probe for *Vmn2r69* (images are representative from a set of 24 sections, from six individuals). lu, VNO lumen; spt, nasal septum. Scale bars represent 100 μm; panels without scale bars have the same magnification as the top rightmost panel. Blue labeling indicates DAPI nuclear staining

ISH experiments performed on VNO sections from mice at different ages showed that the *Olfr692* gene is expressed in a very small subpopulation of cells in juvenile mice at ages P0, P10 and P20, and in young adult mice at P30 (Fig. 3a). In contrast, the expression increases substantially in older adult animals (P60 animals in Fig. 3a, and animals older than 3 months in Fig. 2a). Such pattern of expression is in striking opposition to the temporal expression of the few other *OR* genes previously studied in the VNO, which have higher expression levels in juveniles (younger than 1 month of age) and very low expression in adults [23]. The expression dynamics of *Olfr692* is also distinct from V2R *VR* genes, which are first observed at embryonic stage E14 [16] and are maintained at high levels throughout postnatal development (exemplified by receptor genes *Vmn2r107* and *Vmn2r69* in Fig. 3b,c).

Together, these data point to *Olfr692* as an *OR* gene expressed in the VNO epithelium in a developmentally regulated manner, with robust expression in adults older than 2 months, suggesting that it may be functionally relevant in individuals at that age.

### Molecular characterization of *Olfr692*-positive cells in the VNO

The ectopic expression of *Olfr692* in the VNO may have one of several meanings: (1) VNO *Olfr692*-positive cells may represent neurons with the typical molecular phenotype of canonical *OR*-expressing cells in the MOE (OSNs) but mis-localized to the VNO; (2) VNO *Olfr692*-positive cells may be regular VSNs, expressing all the typical molecular markers of such chemosensory cell type, but with the aberrant (and possibly non-functional) expression of an *OR* gene; (3) *Olfr692*-expressing cells may represent a completely novel subpopulation of sensory cells in the VNO, with its own unique molecular phenotype. In order to discriminate between these possibilities, we set out to molecularly characterize the cells that express *OR* genes in the adult VNO by double fluorescent ISH with cell type-specific markers.

OSNs, but not VSNs, express the olfactory Gαolf G protein subunit [24], thought to couple with adenylyl cyclase subtype III and promote the opening of a cyclic-nucleotide gated channel (CNGA). CNGA is composed of several subunits, one of which, CNGA2, is characteristic of OSNs [25].

In contrast, VSNs in the apical zone of the VNO express V1R family receptors, the G protein Gαi2 subunit and the transient receptor potential family member C2 (TrpC2) ion channel. Each VSN in the basal zone expresses one member in clades A, B or D of V2R receptors combined with the expression of clade C V2Rs, along with the G protein Gαo subunit and TrpC2; some basal zone neurons also express MHC class I H2-Mv family molecules [26, 27].

**Fig. 4:**
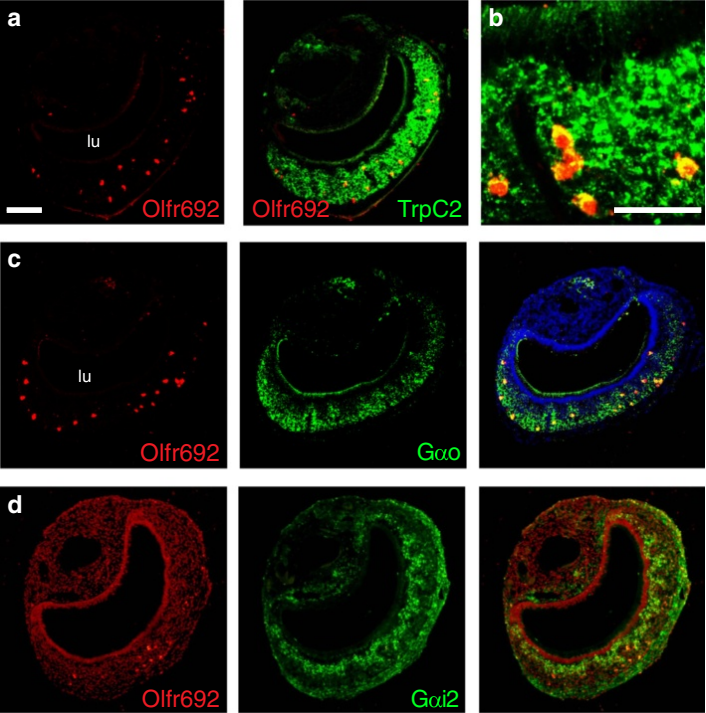
*Olfr692*-positive cells co-express *TrpC2* and are located in the basal layer of the vomeronasal organ. (**a**, **b**) VNO sections subjected to double fluorescent in situ hybridization (ISH) showing co-expression of *Olfr692* (red) and *TrpC2* (green), coding for an ion channel characteristically expressed in vomeronasal neurons (higher magnification image in **b**). (**c**, **d**) Double fluorescent ISH to evaluate expression of genes for G protein subunits Gαo (**c**), a basal VNO zone marker, or Gαi2 (**d**), an apical zone marker, in *Olfr692*-expressing VNO cells. Number of imaged sections and quantification of co-labeling counts are summarized in Additional file 10: Table S2. lu, VNO lumen. Scale bars represent 100 μm and are the same as in the left top panel, except in **b**. Nuclear To-Pro-3 labeling is light red in **d** and blue elsewhere

We found that *Olfr692*-positive cells in the VNO co-express the TrpC2 ion channel (Fig. 4a,b). *TrpC2* is also expressed in a restricted subset of molecularly atypical OSNs [28], but these do not express *Olfr692* (Additional file 3: Figure S2a), supporting the notion that *Olfr692*-positive cells in the adult VNO and MOE are molecularly different. Moreover, we found no evidence of CNGA2 and Gαolf expression in the VNO, though both are abundant in the MOE (Additional file 3: Figure S2b–d). Together, these data show that VNO cells expressing *Olfr692* are not merely misplaced OSNs, because they do not express the full complement of OSN markers.

We found that *Olfr692*-positive vomeronasal cells express Gαo, but not Gαi2 (Fig. 4c,d), consistent with their position in the basal layer of the VNO. It is important to note that such data are to be taken only as part of a molecular characterization of *Olfr692*-expressing VNO cells, not necessarily implying that the corresponding OR receptor couples with Gαo.

Since *Olfr692*-expressing cells are located in the basal zone of the VNO, we sought to determine whether they co-express genes for VRs in the V2R family, which are known to be restricted to the basal layer [16]. If we found evidence for co-expression with *V2R*s in these cells, it could in principle suggest that they are canonical VSNs with aberrant expression of one *OR* gene. In double ISH experiments, we found no substantial overlap between *Olfr692* and staining with probes designed to subclades A1, A2, A3, A4, A5, A8, A9, B, and D of V2R receptors (Additional file 4: Figure S3; Additional file 5: Figure S4a–c), which represent >98 % of all V2R receptors expressed singularly in basal zone VSNs [29] (see also Additional file 6: Figure S5 and Additional file 2: Dataset S1 for probe details and validation). These results strongly argue against the hypothesis that *Olfr692*-expressing VNO cells are canonical VSNs with aberrant *OR* expression.

Basal layer VSNs also express V2R receptors in clade C, which are combinatorially co-expressed with clade A/B/D receptors [30, 31]. We identified a clear co-localization of *Olfr692* with *Vmn2r2* and partial co-localization with *Vmn2r1* (Additional file 5: Figure S4d,e), which are the two clade C members most widely expressed in the VNO [31]. Interestingly, we observed no or little overlap between the expression of *Olfr692* and members of the H2-Mv family of non-classical class I MHC molecules (Additional file 5: Figure S4f,g), which are expressed in a large number of basal zone VSNs [26, 27].

Taken together, the experiments above show that *Olfr692* is expressed in a subset of basal VNO zone cells that are neither canonical VSNs nor misplaced OSNs. Instead, they co-express a unique set of molecular features (TrpC2/V2R clade C/Gαo-positive and CNGA2/V2R clades ABD/Gαolf-negative) that suggests they represent a distinct and novel subpopulation of chemosensory cells in the adult mouse vomeronasal system.

### Investigation of activity in *Olfr692* cells after exposure to various odorous stimuli

To identify a source of ligands able to activate *Olfr692*-expressing cells, we first exposed adult animals to biologically relevant stimuli and then labeled the activated neurons on VNO sections by double ISH with probes for *Olfr692* and for the surrogate marker of VNO neuronal activation *Egr1* [32].

**Fig. 5:**
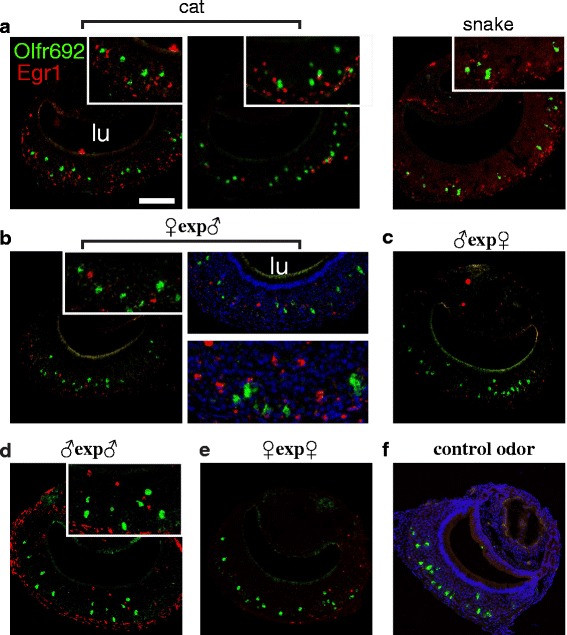
*Olfr692* cells are not activated by predator odors nor by same- or opposite-sex conspecific individuals. Double fluorescent in situ hybridization for the marker of vomeronasal neuron activation *Egr1* (red fluorescence) and *Olfr692* (green) in animals exposed to a range of biologically relevant stimuli: (**a**) Adult male C57BL/6 mice exposed to heterospecific stimuli from cat (left) or snake (right), which have been shown to robustly activate the VNO [32] and to elicit defensive behaviors [14]. Insets are higher magnification images of the corresponding panels to evidence absence of co-expression of *Egr1* and *Olfr692*. (**b**–**e**) Adult C57BL/6 mice exposed to same-strain male or female adult mouse odors. (**b**) In female mice exposed to scented bedding containing male odors and in male mice exposed to the odors of (**c**) female or (**d**) male adult mice, a number of VNO cells are activated, but none overlap with the expression of *Olfr692*. (**e**) In female C57BL/6 mice exposed to female odors, activated cells are virtually absent in the VNO. (**f**) Control animal exposed to unscented bedding. Similar results are obtained upon exposure to clean gauze. Microscopy images are representative from the set of scored sections indicated in Additional file 7: Table S1. See quantification of *Egr1/Olfr692* co-labeling counts in Additional file 7: Table S1. lu, VNO lumen. Scale bars represent 100 μm. Nuclear staining is To-Pro-3 labeling (blue)

Exposure of adult C57BL/6 animals to heterospecific stimuli (odors from predatory species, such as felines, snakes and birds of prey) resulted in no activation in *Olfr692*-positive cells (Fig. 5a; see also [14, 32]), even when these stimuli were used at high quantities, sufficient to activate the VNO maximally (see Methods for amounts of stimuli used and exposure protocols); likewise, no activity was seen upon exposure to adult male and female conspecific odors (Fig. 5b–e), nor to purified aliphatic acid odorants known to be detected by OR receptors similar to Olfr692 [33] (see Additional file 7: Table S1 for quantification of activation in *Olfr692* cells, and Additional file 8: Figure S6a,b).

Additionally, we conducted a comprehensive in vitro screening among a variety of 70 organic candidate Olfr692 ligands (Additional file 8: Figure S6c). Nine chemicals generated responses that were initially suggestive of receptor-mediated activation in *Olfr692*-expressing Hana3a heterologous cells (Additional file 8: Figure S6c; [34]), but on further investigation we found no evidence of statistically significant dose–dependent responses (Additional file 8: Figure S6d).

These experiments suggest that the function of *Olfr692*-expressing cells may not be related to the generation of known VNO-mediated defensive, sexual, or territorial behaviors [7–9, 14].

### *Olfr692*-expressing cells are activated by pup odors

Pup odors have recently been shown to activate vomeronasal neurons in vivo [34], possibly mediating behaviors towards the young. Since *Olfr692* is expressed at very low levels during development and infancy but possesses robust expression in adults above the age of 2 months, we hypothesized that the *Olfr692* cells may mediate chemical communication between juveniles and adults.

**Fig. 6:**
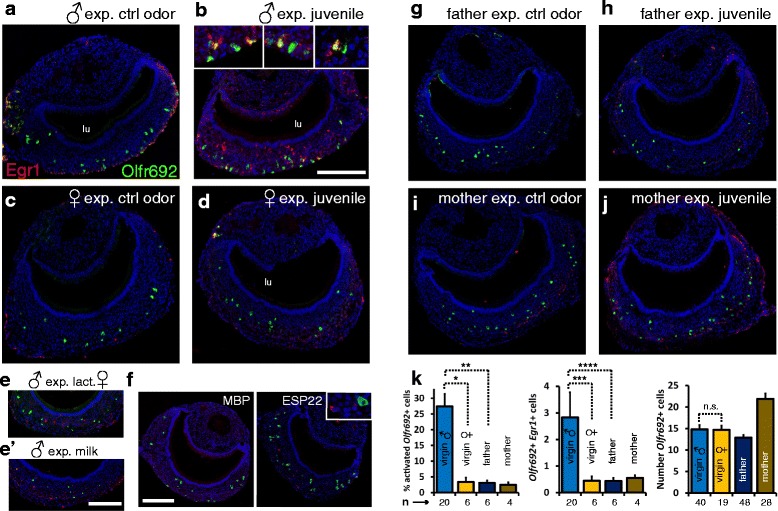
*Olfr692* cells are activated by juvenile odors in a sexually dimorphic manner and depending on the individual’s social status. (**a**–**j**) Double fluorescent in situ hybridization for receptor gene *Olfr692* (green) and immediate-early gene *Egr1* (red), marker of vomeronasal neuron activation, in adult C57BL/6 virgin males (**b**), virgin females (**d**), parenting fathers (**h**) and mothers (**j**), exposed to same-strain pup. For **h** and **j**, exposure was to alien pups. Insets in **b** show higher magnification images of activity in *Olfr692* cells in virgin males. Virgin males, virgin females, fathers and mothers were also exposed to control odors (**a**, **c**, **g** and **i**). Lactating female C57BL/6 mice (**e**) or milk extracted from lactating females (**e'**) activate the VNO of adult male mice (*Egr1* staining in red), as compared to control non-exposed animals (**a**). However, activated cells do not express *Olfr692* (green fluorescence). (**f**) *Egr1* expression (red) shows that recombinantly expressed ESP22 juvenile peptidic pheromone does not activate *Olfr692* cells (green) in adult C57BL/6 males (right); exposure to MBP, the purification label for ESP22, does not activate the VNO (left). (**k**) Quantification of labeled cells in experiments shown in **a**–**h**. Left panel: percentage of *Olfr692* labeled cells per section that are also *Egr1*-positive (n is the number of mice; * *P* = 0.0001, two-sample Welch’s *t*-test assuming unequal variances, *t* = 5.136; ** *P* = 0.005, Welch’s *t*-test, *t* = 4.407; see also Additional file 7: Table S1 for percentage of *Egr1*-positive cells among all counted *Olfr692* labeled cells). Middle panel: number of *Egr1*/*Olfr692* doubly labeled cells per VNO section (n is the number of mice; *** *P* = 0.02, two-sample Welch’s *t*-test, *t* = 2.474; **** *P* = 0.02, Welch’s *t*-test, *t* = 2.509). Right panel: total number of *Olfr692*-positive cells per VNO section (n is the number of sections; n.s. = statistically non-significantly different, two-sample *t*-test, *P* = 0.461, *t* = 0.099). Microscopy images are representative from the set of scored sections indicated in Additional file 7: Table S1. Quantification of activation in all experiments is detailed in Additional file 7: Table S1. lu, VNO lumen. Scale bar = 100 μm. In graphs, mean ± SEM

**Fig. 7:**
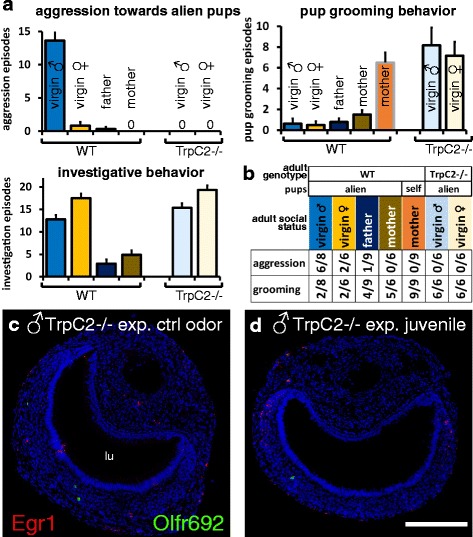
Activation of *Olfr692* cells in the VNO of virgin male mice correlates with pup-directed aggressive behavior. (**a**) Juvenile-oriented aggressive behavior (top, left) and parental care behavior (pup grooming; top, right) exhibited by virgin females, virgin males, parenting males (fathers), and parenting females (mothers) of wild-type (WT) C57BL/6 strain, showing pup-directed aggression in naive males (n = 6–9 mice). In the absence of a functional VNO (*TrpC2*-/- mutant mice), infanticidal behavior is impaired and the expression of parental care (pup-grooming) is similar to that exhibited by mothers. Bars with black outlines represent individuals exposed to alien pups; gray outlines indicate exposure to self-pups. Investigative behavior is also shown (bottom, left). ‘0’ indicates no episodes of the measured behavior. (**b**) Number of animals which exhibited at least one episode of the measured behavior (pup-directed aggression or grooming) in wild-type (WT) C57BL/6 or *TrpC2*-/- animals. ‘1/9’ indicates that one animal displayed the behavior in a sample of n = 9. (**c**, **d**) *Olfr692* cells are rarer in the VNO of *TrpC2*-/- mice and are not activated by juvenile odors (**d**). lu, VNO lumen. Scale bar = 100 μm. In graphs, mean ± SEM

We assayed the activation of *Olfr692* cells in adult C57BL/6 mice exposed to pup odors, in several behavioral contexts; each exposure was to one or two C57BL/6 same-strain juvenile mice (P0.5–P8.5; male or female), for 45 min. Because father and mother mice are constantly exposed to pup odors, they could show desensitization of OSNs tuned to detect juvenile stimuli. Therefore, we first exposed virgin (nulliparous) adults to alien juveniles. Strikingly, when sexually naive (virgin) adult males were exposed to same-strain (C57BL/6) pups (P0–P8.5), they showed a very robust activation of *Olfr692*-positive VNO cells (27.4 ± 4.0 % of all *Olfr692* cells per section; n = 20 mice, two sections per individual; Fig. 6b,k). In striking contrast, when nulliparous (virgin) females were exposed to same-strain juveniles of either sex, virtually no *Olfr692*-positive VNO cells were activated (Fig. 6d,k; *Egr1* counts in Additional file 7: Table S1), even though the females actively investigated the juvenile subjects (Fig. 7a). Importantly, the number of *Olfr692*-expressing cells in wild-type C57BL/6 mice is equivalent in virgin males [14.8 ± 1.0 *Olfr692*-positive cells per section (n = 40 sections, two sections per individual)] and virgin females [14.7 ± 1.0 (n = 19 sections, three to four sections per individual)] (Fig. 6k, right graph). Together, these data show that VNO cells that express *Olfr692* are activated differently in males and females.

To ensure that the activation seen in males exposed to alien pups is not due to odors derived from the pup’s mothers, we exposed C57BL/6 male mice to lactating females or to scented bedding of lactating females. We observed only a very limited number of cells activated in the VNO, and these showed very little overlap with the expression of *Olfr692* (Fig. 6e). Similarly, although we observed a small subpopulation of cells in adult males activated by milk extracted from lactating females, these cells do not overlap with those expressing *Olfr692* (Fig. 6e’; Additional file 7: Table S1).

Moreover, to ensure that activation in *Olfr692* cells is not due to odors released by the pup as a consequence of its interaction with the adult male, nor due to chemosignals detected by the adult during the act of biting the pup (blood-derived ligands), we exposed male adults to ligands washed off the pups (pup wash) and to pups placed inside a plastic capsule bearing 4-mm wide holes, which allowed the adult to approach the pup without being able to bite it. In both cases, we observed robust activity in *Olfr692* VNO neurons (Additional file 9: Figure S7; see Methods section for details). These data prove that aggressive pup-adult physical interaction is not needed for the pup-emanating odors to activate *Olfr692* cells in the adult VNO (see also [35]).

Together, we confirm that the VNO is capable of detecting odors from young conspecific individuals [35], and show that the *Olfr692*-expressing neural subpopulation is involved in this detection process. Even though the *Olfr692* neurons are numerically equivalent in both sexes, their response to pup odors is sexually dimorphic. This is, to our knowledge, the first documented case of sexual dimorphism in the activity of a mammalian chemosensory subpopulation expressing a defined receptor.

### Activation of *Olfr692*-expressing cells depends on the animal’s social status

A number of pup-oriented behaviors may be triggered by the VNO. A recent study has shown that the VNO mediates the inhibition of adult sexual behavior towards pups via detection of the ESP22 peptide, secreted by the lacrimal gland of juveniles [12]. However, we did not detect activation of the *Olfr692*-positive VNO population in adult animals after exposure to the recombinant version of ESP22 swabbed onto a juvenile from the C3H strain (which does not express ESP22 naturally) (Fig. 6f).

Other studies reported that the VNO is required for parental behaviors [36]. When we exposed parenting males (fathers) to C57BL/6 alien pups (juveniles collected from other breeding cages), we observed very little activation of *Olfr692*-expressing cells (3.1 ± 0.8 % of all *Olfr692*-positive cells per section; n = 6 mice, eight sections per mouse; Fig. 6h,k), in contrast to the robust percentage of activated *Olfr692* cells (27.4 ± 4.0 %) observed in virgin, sexually naive male individuals (Fig. 6b). Even though activity is distinct between virgin and parenting males, the number of *Olfr692* cells in fathers (12.9 ± 0.6 *Olfr692*-expressing cells per section; n = 48 sections, eight sections per individual) is similar to that seen in virgin males (Fig. 6k, right graph). Strikingly, these results suggest that the activity in the adult VNO *Olfr692* population is dependent on the animal’s social/parenting status.

In mothers, the overall number of activated cells in the VNO when animals were exposed to alien pups was appreciable (Fig. 6j), but the overlap with the expression of *Olfr692* was very small (2.5 ± 0.8 % of all *Olfr692*-positive cells per section; n = 4 mice, six to eight sections per mouse). Interestingly, we found more *Olfr692*-positive cells in mothers than in males or virgin females (21.9 ± 1.3 *Olfr692*-positive cells per section; n = 28 sections, six to eight sections per individual; Fig. 6k, right graph).

Finally, we investigated possible behavioral consequences of *Olfr692*-expressing cell activation in the VNO. We found that the high activity in *Olfr692* cells in virgin males is positively correlated with the display of extremely potent aggressive behavior and absence of pup-retrieval parenting behavior towards alien juveniles (Fig. 7a,b). In contrast, females and parenting males, in which activation of *Olfr692* cells is low, do not exhibit aggressive bouts to alien pups (Fig. 7a,b; see also [36–40]). The correlation between high activity in *Olfr692*-positive VNO cells and the display of pup-oriented aggression together with absence of pup-directed care suggests that such sensory population may be involved in mediating parental behaviors. This possibility would be further strengthened by the observation that mice without a functional VNO show impairment in these behaviors. In fact, in our paradigm, we observed changes in pup-directed behaviors, such as impaired pup-directed aggression and more pup retrieval parenting behavior, in animals with genetic ablation of the VNO by a null mutation in the *TrpC2* gene (*TrpC2*-/-; Fig. 7a,b). Additionally, we saw no activation of *Olfr692*-expressing VNO neurons in *TrpC2*-/- sexually naive adult males exposed to wild-type alien pups (Fig. 7d), in contrast to the robust activity seen in wild-type adult males (Fig. 6b). Similar behavioral results were obtained by groups working with sexually naive males subjected to surgical removal of the VNO [35] or with VNO genetic ablation [36].

Together, our data show that *Olfr692*-positive cells are highly activated by pup odors in infanticidal virgin wild-type males, while activity and behavior are lost when the VNO is held non-functional by genetic ablation. We hypothesize that the pup odor-responsive sensory population expressing the *Olfr692* gene mediates pup-oriented behaviors like infanticidal aggression or parental care. In the future, further experiments on *Olfr692*-/- knockout mice will allow us to test this hypothesis.

## Discussion

In this paper, we describe an atypical subpopulation of neurons in the VNO, one of the olfactory sensory structures, that expresses a specific gene in the OR family, *Olfr692*. We found an extensive number of *Olfr692*-positive cells in VNOs from adult mice, but expression is virtually absent in juveniles (Figs. 1, 2, 3). This expression pattern is distinct from those of *VR* genes, which are first expressed in embryos [15, 16], and is also different from the few *OR*s reported in the VNO, which are mostly expressed in juveniles [23]. Moreover, *Olfr692*-expressing cells are Gαolf/CNGA-negative, TrpC2/Gαo-positive, and located in the basal zone of the VNO. The robust expression of *Olfr692* in the adult VNO and co-expression with specific molecular markers suggest that the VNO sensory population expressing such gene is distinct from previously described VSN categories.

*Olfr692* is, to our knowledge, the first OR gene shown to be expressed almost exclusively in adults, suggesting a critical role at this stage in the life cycle. Interestingly, *Olfr692* is differentially expressed between the two main olfactory sensory organs, having a very low expression level in the MOE, but a surprisingly high expression level in the VNO (Fig. 1), suggesting that the *Olfr692*-expressing population modulates instinctive behaviors mediated by the VNO.

Which stimuli activate the *Olfr692*-expressing VNO population? Known vomeronasal ligands include urine-derived small organic molecules [20, 41], sulfated steroids [32, 42], MHC peptides [43], ESP peptides [12, 13], and conspecific or heterospecific small proteins [8, 14]. Moreover, a fraction of VNO neurons respond to small organic volatile odorants in vitro [20, 41]. It is possible that such detection may involve the cells expressing *Olfr692*, since the OR family is traditionally associated with the detection of volatile odorants.

In this paper, we have evaluated the activation of the *Olfr692*-positive VNO population after exposure of animal subjects to biologically relevant odor sources. We show that *Olfr692*-positive cells in the adult VNO are activated by pup odors (Fig. 6). Strikingly, this activation is sexually dimorphic: even though the number of *Olfr692*-positive cells is equivalent in males and females, the number of activated cells in virgin females is very low, while virgin males exhibit robust activation of *Olfr692*-positive cells after exposure to pups (Fig. 6). This is the first reported case of sexual dimorphism in activity of a sensory population expressing a specific olfactory receptor in mammals.

Additionally, we show that mothers and fathers have surprisingly low activation of *Olfr692*-expressing neurons overall, even though cells expressing this receptor are present in equivalent numbers in virgin and parenting individuals (Fig. 6). In comparison with the large number of active cells seen in virgin males, these results indicate that activity depends on the animal’s social and parenting status, probably modulated at the sensory interface by the individual’s internal hormonal state. A similar phenomenon was recently observed for another subgroup of VSNs, involved in detecting odors from opposite sex individuals, which was reported to be modulated by steroid hormones in mice [44].

Which behaviors might be mediated by the *Olfr692*-expressing population? The VNO mediates a range of instinctive behaviors, including individual identity recognition [45], aggression [8], and interspecies communication [14]. Since *Olfr692* is expressed differentially between juveniles and adults, it is conceivable that it mediates adult-specific behaviors. We did not find evidence of involvement in sexual behaviors (Fig. 5); instead, we suggest that *Olfr692*-expressing cells mediate adult behaviors towards the young.

We found robust activation of *Olfr692*-positive VNO cells in sexually naive male adults exposed to alien pups (Fig. 6), a context in which strong aggressive behavior is displayed towards juveniles (Fig. 7). Interestingly, little discernible activation of *Olfr692* cells is found in virgin females exposed to alien pups or in mothers and fathers (Fig. 6), where aggression is not present (Fig. 7). The possibility that *Olfr692*-positive vomeronasal neurons are involved in aggressive behavior towards pups finds support in previous data showing that the VNO detects juvenile odors and mediates infanticidal behavior [35, 36]. Infanticide has been documented in virgin male mice of many strains, including C57BL/6 (up to 70 % infanticide rate) [39], and this phenomenon has been suggested to be adaptive because survival of a male’s own biological offspring at the expense of alien, potentially competing, offspring is evolutionarily beneficial.

Alternatively, *Olfr692*-expressing VNO neurons might mediate the inhibition of parenting behaviors in virgin males. Interestingly, males with surgical or genetic ablation of the VNO exhibit significantly more episodes of pup retrieval, pup grooming and nest building than control animals [35, 36].

Because we observed robust activity in *Olfr692*-positive neurons in non-fathers but very low activity in parenting fathers (Fig. 6), a third possibility is that the *Olfr692*-expressing neurons mediate the interplay between aggressive and parental behaviors; this model concurs with the observation that aggressive C57BL/6 virgin males exhibit a switch to parenting behaviors after sexual experience, even towards alien pups [40]. Such behavioral switch has been hypothesized to be mediated by olfaction [46]. Moreover, manipulation of activity in neurons in the medial preoptic area or the bed nucleus of the stria terminalis in the brain, which receive functional inputs from the VNO, has been shown to lead to a switch between aggressive and parenting behaviors in males and females [35, 36, 47].

Further investigation on knockout mice carrying null mutations in the *Olfr692* gene will be important to provide evidence that the Olfr692 receptor detects pup odors, and to elucidate the nature of the behavior mediated by the *Olfr692*-expressing sensory neurons.

## Conclusions

Most animal species use olfaction to appropriately perceive and respond to the external world. Among the many detected odors are those able to elicit instinctive behaviors, such as pheromones. Knowledge on the mechanisms animals use to sense such cues is key to understanding animal behavior, population dynamics, life cycle, natural history, and evolution. Here, we described a non-canonical subgroup of OSNs, located in the specialized VNO. These neurons unusually express a receptor gene in the OR family, which is typically found in another olfactory structure in the nose, the main olfactory epithelium. These cells seem to be non-canonical because they express a unique complement of molecular markers. Importantly, we found that pup odors activate these neurons. Moreover, activation is sexually dimorphic, because it is seen only in adult males exposed to pups, a rare case of sex difference in olfactory organ activation. Strikingly, activity also depends on the animal’s social and parenting status: robust activity is seen only in virgin adult males exposed to pups, a situation in which potent aggressive (infanticidal) behavior is triggered, and not in parenting adults.

We anticipate that our findings will shed light onto some outstanding questions in sensory biology, including the molecular details of how pup-derived odors are recognized by specific receptors to trigger instinctive responses in adults. Although the involvement of olfaction and the vomeronasal system in parental behaviors has been previously suggested, no specific population of pup-detecting olfactory cells had been identified, a gap of knowledge that is now filled by the present study. Once the participation of the *Olfr692* population in the regulation (triggering/inhibition) of pup-oriented behaviors in adults is established, pup-derived olfactory cues purified based on our work will be important to better study this behavioral phenomenon. How do the *OR*-expressing cells mediate the interplay between parental and infanticide behaviors? Why is infanticide common in so many animal species? How important is olfaction in the generation of such responses? Our findings may fuel future research to understand the molecular and neural mechanisms behind these questions.

## Methods

### Mice

Animals were 2- to 4-month-old male mice, unless otherwise noted. *TrpC2*+/+ and *TrpC2*-/- littermates were obtained from heterozygous mating couples, which were produced by backcrossing the *TrpC2*-/- knockout line [9] into the C57BL/6 background for at least 10 generations. Juveniles used in Figs. 6 and 7 were P0.5 to P8.5 C57BL/6 pups, except in Fig. 6f, which required the use of pups in the C3H strain. Animals used in this study were obtained directly from our vivarium facility, and procedures were carried out in accordance with Animal Protocol no. 1883-1, approved on June 2009 by the Institute of Biology’s Institutional Animal Care and Use Committee (Committee for Ethics in Animal Use in Research), at the University of Campinas. This protocol follows the guidelines established by the National Council for Animal Experimentation Control (CONCEA-Brazil).

### RNA sequencing and gene annotation

Whole adult VNO and MOE RNA sequencing have been previously described [21]. For the newborn juvenile data, the whole VNO was dissected from P0.5 C57BL/6 J animals (of both sexes) and the tissue from three to four animals was pooled for each sample and stored in RNAlater (Qiagen). RNA was extracted using the RNeasy mini kit (Qiagen) with on-column DNAse digestion. A fragment range of 200–300 nt was selected from mRNA prepared for sequencing using the TruSeq RNA sample preparation kit (Illumina). Samples were multiplexed together and sequenced on one lane on the Illumina HiSeq 2000 to generate 100 bp paired-end reads; on average, each sample yielded 59.85 ± 6.02 million fragments. Data processing was done as previously published [21]. Briefly, reads were aligned to the GRCm38 mouse reference genome and the number of fragments uniquely aligned to each gene was counted using the HTSeq package; the annotation used was from the *Ensembl* mouse genome database, version 68 (http://jul2012.archive. ensembl.org/info/data/ftp/index.html). Counts were then normalized for depth of sequencing and gene length to obtain FPKM values [48]. Multi-mapping reads were not included in the FPKM calculations. Sequencing data are available in the European Nucleotide Archive under accession PRJEB1607.

### TaqMan qRT-PCR

RNAs from MOE and VNO were extracted from four individual male, 8-week-old C57BL/6 J mice. TaqMan probes were used to assess gene expression of target *OR* genes, which was performed on a 7900HT Fast Real-Time PCR System (Life Technologies) according to the manufacturer’s instructions. Mean cycle threshold (Ct) values from three technical replicates were each normalized to β-actin expression, using the ΔCt method. Relative quantity (RQ) values were calculated using the formula RQ = 2^-ΔCt^. The TaqMan probes used were: Mm00529996_s1 (Olfr124), Mm00526312_s1 (Olfr692), Mm00451556_s1 (Olfr1509), Mm00498747_s1 (Olfr1264), Mm00729160_s1 (Olfr1512), Mm02015876_s1 (Olfr1372-ps1), and Mm00453733_s1 (Olfr78).

### In situ hybridization (ISH)

For the design of cRNA probes to *V2R* VR genes, we investigated whether different genes harbor specific regions anywhere in the coding or non-coding regions. However, nucleotide and protein similarities among the members of each clade were found to be very high (>80 %), though members from different clades usually share less than 75 % nucleotide sequence identity (see also Additional file 2: Dataset S1; and Additional file 6: Figure S5). These similarity levels are constant throughout the entire gene sequence, including exons, introns, and untranslated regions. For each clade, we chose probes based on one or two receptors. For each *V2R* gene, a probe (length 1–1.2 kb) was designed based on the 1.5 kb region starting at the translation initiation codon. Each *V2R* receptor probe consistently labels the same subset of vomeronasal neurons, as judged by co-staining with probes for the same receptor labeled with different haptens (Additional file 6: Figure S5a). Usually, a probe based on one *V2R* gene in a particular subclade A was able to label most VSNs expressing receptors in that subclade if they all share more than 80 % similarity (Additional file 6: Figure S5b–d). Occasionally, two probes were used to cover cells expressing all receptors in a subclade, if some of its receptors share less than 80 % similarity (Additional file 6: Figure S5e). Probes for distinct subclades in clade A do not produce significant overlap (Additional file 6: Figure S5f,g). We used distinct probes for each member in the V2R C family. For the neuronal marker *Egr1*, we used three non-overlapping 1-kb probes spanning the entire coding sequence, as previously published [32]. Oligonucleotides used as primers to amplify these and other target genes can be found in Additional file 2: Dataset S1.

Each cRNA probe was produced with rNTPs labeled with haptens DNP, FLU, and/or DIG (Roche) from fragments cloned in pGEM-T-Easy vector (Promega), using SP6 or T7 RNA polymerases (Roche). Slides containing 16-μm cryostat coronal VNO sections were air-dried for 10 minutes, followed by fixation with 4 % paraformaldehyde for 20 min, and treated with 0.1 M HCl for 10 min, 0.1 % H_2_O_2_ for 30 min, and 250 mL of 0.1 M triethanolamine (pH 8.0) containing 1 mL of acetic anhydride for 10 min, with gentle stirring. Slides were always washed twice in 1× PBS between incubations. Hybridization was then performed with DNP (1 μg/mL) or DIG (600 ng/mL) labeled cRNA probes at 58 °C in hybridization solution (50 % formamide, 10 % dextran sulfate, 600 mM NaCl, 200 μg/mL yeast tRNA, 0.25 % SDS, 10 mM Tris-HCl pH 8.0, 1× Denhardt’s solution, 1 mM EDTA pH 8.0) for 16 h. Slides were washed once in 2× SSC, once in 0.2× SSC and once in 0.1× SSC at 60 °C (30 min, 20 min and 20 min, respectively), followed by a quick incubation in 0.1× SSC at room temperature. Slides were then permeabilized in 1× PBS, 0.1 % Tween-20 for 10 min, and washed twice in TN buffer (100 mM Tris-HCl pH 7.5, 150 mM NaCl) for 5 min at room temperature, followed by blocking in TNB buffer (100 mM Tris-HCl pH 7.5, 150 mM NaCl, 0.05 % blocking reagent (Perkin Elmer)), and incubation overnight at 4 °C with rabbit anti-DNP (Invitrogen) primary antibody diluted 1:600 in TNB buffer. Signal development proceeded with the tyramide signal amplification kit (Perkin Elmer), following the manufacturer’s instructions. Briefly, slides were incubated in tyramide-biotin (1:50 in amplification diluent with 0.0015 % H_2_O_2_ (Perkin Elmer)) for 15 min, followed by incubation in streptavidin-HRP (1:100 in TNB) for 1 h and incubation in tyramide-Alexa 546 (1:100 in amplification dilutent with 0.0015 % H_2_O_2_ (Life technologies)) for 15 min. Prior to each incubation, slides were washed six times with TNT buffer for 5 min under mild agitation. Sections were then treated with 3 % H_2_O_2_ in 1× PBS for 1 h to block peroxidases from the first signal development. Slides were then blocked in TNB for 90 min, followed by incubation overnight at 4 °C with anti-DIG-POD (Roche) diluted in TNB (1:400). Signal development for DIG probe was performed using tyramide signal amplification kit (Perkin Elmer) and tyramide-Alexa 488 dye. Samples were counter-stained with To-Pro 3 nuclear stain (Invitrogen) diluted 1:1,000 in 1× PBS, washed twice in 1× PBS and mounted with ProLong Gold (Invitrogen).

For counting cells in the apical or basal zones of the VNO (Fig. 2e and Additional file 1: Figure S1f), a line dividing the VNO epithelium in half was manually drawn onto each VNO section used for counting and the position of each stained cell was computed. This approach ignores the fact that the real dividing line between both zones is circumvoluted, but was used here as a first approximation, in combination with double ISH experiments with G protein zonal markers.

For some double ISH, we combined chromogenic in situ detection for receptor genes (V2R receptors in Additional file 4: Figure S3; Additional file 5: Figure S4a,b, and *Egr1* in Fig. 6e’) with fluorescent in situ detection for *Olfr692*, because chromogenic development is more sensitive, ensuring that the full complement of positive cells was unequivocally labeled. Since the chromogenic stain may cause blocking and quenching of underlying fluorescence, we used confocal microscopy to make sure that all fluorescently-labeled neurons were visualized, even in the presence of purple precipitate from the chromogenic detection phase. Moreover, we conducted a control experiment with two probes for the same gene, visualized concomitantly with fluorescent and chromogenic ISH, to confirm that no quenching of fluorescence was occurring due to the colored precipitate from chromogenic detection (Additional file 5: Figure S4c). The image shown in Fig. 6e’ is false colored to represent the chromogenic signal as red and *Olfr692* fluorescence as green.

For double *Egr1/Olfr692* ISH experiments, it is important to note that the number of *Egr1*-positive cells is to be taken as the minimum estimate of the real number of active *Olfr692* cells, because *Egr1* is a surrogate marker of vomeronasal neuron activation, and its mRNA appears in the cell body’s cytosol during the 20–50 minute window after the onset of ligand detection, being completely degraded after 1 h from the onset of stimulation. Therefore, it is possible that some *Olfr692* cells may have detected its ligand at the beginning of the 45 min exposure period, resulting in robust expression of *Egr1* at the time the animals were sacrificed; other *Olfr692* cells, on the other hand, may have detected the stimulus at the end of the exposure window and therefore did not have enough time to express *Egr1* mRNA. Moreover, the *Olfr692* sensory subpopulation may be composed of mature and immature cells, and since we are detecting the expression of *Olfr692* mRNA, it is possible that not all *Olfr692*-positive cells are functionally mature and capable of detecting the pup stimuli. These arguments indicate that the real percentage of *Olfr692* cells activated by pup odors may be higher than the percentage estimated by *Egr1* staining.

### Stimuli

Cat odor was obtained by rubbing a medical gauze against the fur of a domestic cat, particularly around the neck region, which is constantly licked by the subject [14]. For snake odor, we used 1 g of stimulus corresponding to around four 5 × 5 cm pieces of shed skin. Fifty milliliters of scented bedding (fine wood chips) were used as male and female mice odor stimuli. Control mice were exposed to clean bedding. For collection of milk, lactating females older than 3 months were anesthetized and injected with 3U oxytocin supplemented with 15–20 μL of 1 mg/mL solution of metochlopramide chloridrate (Maxeran; Sanofi-Aventis). Subjects were positioned belly up and the two front nipples were connected to a hand-made suckling device controlled by a vacuum source. Milk collected on the tubing was dried and rehydrated in 0.5 M EDTA in 1× PBS to create a 1:2 milk suspension. Subjects were exposed to 500 μL of this solution, and controls were exposed to EDTA solution alone. Octanoic, nonanoic, decanoic, and stearic acids (Sigma) were prepared as 100 mM stocks in mineral oil, and subjects were exposed to 500 μL of either a 1 mM or a 100 mM solution prepared in the same vehicle. For ESP22 peptide, the subject was exposed to 15- to 18-day-old juveniles from the C3H strain that had been swabbed with 10 μg of recombinant protein (as fusion with maltose-binding protein, MBP) on its back; C3H juveniles swabbed with MBP or C57BL/6 juveniles were used as control stimuli. In Figs. 6 and 7, we used C57BL/6 3- to 4-month-old mice exposed to one or two P0.5–P8.5 C57BL/6 pups for 45 min. Most of these experiments were conducted with P0 juveniles, where sexing by visual inspection is difficult; therefore, male and female newborns were chosen at random from a large set of pups from different breeding cages. For experiments in Additional file 9: Figure S7a, pup wash was collected by placing two P0.5–P2.5 C57BL/6 pups head-up in a 50 mL conical tube containing ~2 mL of warm 1× PBS, for 30 min, followed by washing the pups with a pipette, avoiding the head area; for each 3-month-old C57BL/6 subject, exposure was to 1 mL of this pup wash solution, deposited on gauze, for 45 min. For experiments in Additional file 9: Figure S7b, two pups were placed inside a 5 × 10 cm plastic capsule bearing 4 mm holes all around, to allow adult animals to approach the pups without biting them; for each 3-month-old C57BL/6 subject, exposure was to two such capsules, for 45 min.

Whenever possible, olfactory stimuli were presented in the same form and amount, such as equal volumes of scented bedding or equal amounts of scented gauze. For all stimuli deposited on gauzes, the gauze was unscented in a desiccator under vacuum overnight before adding the stimulus. All stimuli (solid or liquid deposited on gauze) were attached to ‘binder clips’ to visually confirm their position and prevent the spreading of stimuli in the cage. Each exposure to potential odor ligands proceeded for 45 min before animals were euthanized for VNO dissection.

### Recombinant ESP peptide expression

The ESP22 peptide expression vector, gently provided by Dr. Stephen Liberles, is based on pMAL-c5x [13], which allows expression of ESP22 as a fusion protein with MBP. Protein was eluted from an amylose affinity resin using maltose and then exchanged into 1× PBS using a YM10 column prior to exposures. Recombinant MBP was used as a control.

### Functional heterologous assays

First, the tested *OR* genes were amplified from genomic DNA and cloned into pGEM-T-Easy Vector (Promega). PCR was then used to insert *Eco*RI and *Not*I restriction sites, which were then used to transfer the *OR* gene to pCI vector (containing the first 20 amino acids of rhodopsin ‘Rho’ tag). Hana3A cells, derived from HEK293, constitutively express RTP1L, RTP2, and REEP1, which are involved in trafficking of the receptor to the extracellular membrane, and Gαolf [24]. In this cell line, ligand detection leads to an increase in the intracellular concentration of cAMP; this messenger is then assayed based on its effect on a second construct bearing a cAMP-responsive promoter driving the expression of secreted alkaline phosphatase (SEAP) [34]. Prior to the experiments, cells were maintained in Minimal Essential Medium with Earle’s Salts supplemented with L-glutamine (Invitrogen), 10 % FBS and penicillin, streptomycin, and amphotericin, and split 1:20 every 3–4 days. Fifty thousand Hana3A cells were placed in each well of a 96-well plate in a total volume of 200 μL MEM with 10 % FBS and, after 24 hours, were transfected with the following plasmids using Lipofectamine2000 (0.3 μL/well in 200 μL MEM; Invitrogen): pCI-Rho-OR (20 ng/well), RTP1S (another accessory factor which aids trafficking of the receptor to the cell membrane, 20 ng/well), and pCRE-SEAP, which expresses secreted alkaline phosphatase using the cAMP-response element promoter (20 ng/well, Clontech). After 18–24 hours, the transfection medium was removed using a multi-channel aspirator, and replaced, for 14–18 hours, with serial dilutions in half-log steps of an odorant diluted with MEM, followed by 2-hour incubation at 70 °C to lyse the cells. The plates were then left to equilibrate to room temperature. Fifty microliters from each well containing cell lysate were transferred to a new 96-well plate, to which 50 μL of 1.2 mM 4-MUP in 2 M diethanolamine were added. The plates were then incubated at room temperature for 30–45 minutes. Fluorescence was measured using a PHERAStar Plus plate reader (BMG Labtech), with a filter unit for an excitation wavelength of 360 nm, and an emission wavelength of 450 nm. In order to compare responses between plates and receptor genes, SEAP fluorescence values were normalized to the fluorescence of wells containing non-stimulated cells (‘fold-change activation’).

### Behavioral analyses

To ensure the identification of instinctive behaviors, animals had no previous exposure to odors from other animal species, and subjects exposed to conspecific chemosignals were kept individually caged for at least 4 days prior to the experiment. All subjects were exposed to odor, monitored for behavior, and subsequently processed for ISH, ensuring that the cellular responses and behaviors were analyzed from the same individuals and no animals were re-used. Wild-type (*TrpC2*+/+) or *Trpc2*-/-, 3- to 4-month-old sexually naive males or virgin, nulliparous, females were exposed to one or two C57BL/6 pups. These pups do not carry scents from the father, because the adult male was removed from the breeding cage prior to pup delivery. Pups were always introduced to the subject’s home cage far from the nesting area. Each behavioral assay proceeded for 5 minutes; when VNOs from the same animals were assayed for activation by ISH, the exposures proceeded for a total of 45 minutes. We scored episodes of pup retrieval, defined as the event in which the animal is carried around by the adult and placed in the nesting area, as well as pup-directed aggression episodes, which were counted based on visual inspection that the pup had been attacked, an event usually accompanied by squeaking. Investigation episodes, including those that led to pup retrieval, were also counted.

### Statistical analyses

Statistical analyses were performed using R and Stat packages, and XLSTAT add-on in Excel. For comparing mean behavioral output measurements, we applied one-way Analysis of Variance (ANOVA), followed by Tukey-Kramer HSD post-hoc analysis. For calculating the mean number of cells per section expressing a certain gene, n equals the number of scored histological sections, which were collected from a certain number of mice (this information is indicated in the text, together with the mean, or in Additional file 7: Table S1 and Additional file 10: Table S2); for cell count scoring, sections were chosen randomly from the set of stained sections. For calculating the mean percentage of *Olfr692*-expressing cells activated by a given stimulus (*Egr1*-positive), n equals the number of mice; for each individual, the percentage count is the mean of all imaged sections, which were randomly chosen among all sections on the slide (see Additional file 7: Table S1 for a complete list of groups analyzed, number of sections scored and number of mice from which these were collected). For comparing mean numbers of *Egr1/Olfr692* doubly-positive cells or the mean percentage of activated *Olfr692*-expressing cells in males and females exposed to pup odors, we applied two-sample Welch’s *t*-test assuming unequal variances. *P* values (probability that the null hypothesis that the means are equal is true) greater than 0.05 led to rejection of the null hypothesis in all tests.

### Availability of supporting data

The newborn olfactory organ RNA sequencing dataset supporting the results of this article is available in the European Nucleotide Archive under accession number PRJEB1607, found at https://www.ebi.ac.uk/ena/data/view/PRJEB1607.

## Additional files

Additional file 1: Figure S1. Investigation of expression of *OR* genes in the VNO by in situ hybridization (ISH) and supporting control data for *Olfr692* expression. (**a**) *Olfr691*, the *OR* gene located closest to *Olfr692* in the mouse genome, is not expressed in the VNO, nor are two other *OR* genes not found in the VNO RNA sequencing library (*Olfr638* and *Olfr569*). The same probes label several neurons in the MOE. Images are representative from sets of 12 sections, from 3 mice. (**b-d**) Chromogenic (left panels) and fluorescent (right) ISH images with probes for V2R receptor genes *Vmn2r41* (**b**), *Vmn2r107* (**c**) and *Vmn2r69* (**d**). See also Additional file 2: Dataset S1 and Additional file 6: Figure S5 for probe validations. (**e**) Chromogenic (left) or fluorescent (right) ISH for *Olfr78* in the VNO (representative from 14 sections, 7 mice). (**f**) Quantitation of location of *Olfr692*- and *Olfr78*-positive cells in the VNO apical (blue bars) or basal (red bars) zones (error bars are SEM; n = 48 sections, 4 sections per mouse, for *Olfr692*; n = 12 sections, 2 sections per mouse, for *Olfr78*). See also Additional file 3: Figure S2e. (**g**) Chromogenic ISH for *Olfr1512* on VNO (left; representative from 27 sections, 3 mice) and MOE sections (right; representative from 24 sections). (**h-j**) Absence of VNO expression for *Olfr124* (**h**, left and middle panels; representative from 32 sections, 16 mice) and *Olfr1509* (**i**; representative from 8 sections, 4 mice). Probe validations in the MOE for *Olfr124* (**h**, right; representative from 12 sections, 6 animals) and *Olfr1509* (**j**; representative from 8 sections, 4 mice). (**k**) *Olfr124* is highly expressed in the Septal Organ of Masera (representative from 12 sections, 4 mice). lu, VNO lumen; ep, MOE sensory epithelium; som, Septal Organ of Masera (SOM); spt, nasal septum. Scale bars represent 100 µm. Nuclear staining is DAPI labeling (blue). (PDF 1.56 MB) Additional file 2: Dataset S1. Pairwise nucleotide sequence similarity for V2R receptors and probe sequence information. ‘V2R pairwise similarity matrix’ spreadsheet: Numbers in each cell represent nucleotide sequence similarity between a pair of V2R sequences in the region where in situ hybridization probes were designed. Similarity levels were calculated from genetic distance matrices produced from multiple alignments generated in MEGA used to construct phylogenetic trees in Additional file 6: Figure S5, according to the formula d = -3/4 ln (1-4/3 D), where d is the genetic distance and D is the fraction of nucleotide sites that differ between the pair of sequences being compared. The receptors are grouped into subclades, shown on the left, according to similarity levels. Triangles of yellow cells indicate comparisons between pairs of V2R sequences in the same subclade. ‘Oligo sequences’ spreadsheet: Sequences of oligonucleotides used as primers to amplify specific regions in target genes to be used for probe synthesis. (XLSX 104 kb) Additional file 3: Figure S2. Control experiments for in situ hybridization investigation (ISH) of the expression of genes for signal transduction molecules in *Olfr692*-positive cells. (**a**) The gene for transient receptor potential member TrpC2, characteristic of VSNs, is expressed in a very limited subset of MOE sensory neurons (chromogenic detection following ISH), but these OSNs do not express *Olfr692*, which would appear as green fluorescent cells in the middle panel (images representative from a set of 21 sections, from seven mice). Blue is DAPI nuclear staining. (**b**) The gene for cyclic nucleotide gated channel subunit cyclic-nucleotide gated channel (CNGA2), characteristic of MOE OSNs (chromogenic ISH in the right panel), is not expressed in the VNO (left). Middle panel shows a higher magnification image of the leftmost panel. For the VNO, images are representative from a set of 12 sections, from six mice; for the MOE, images are from a set of 12 sections, from three mice. (**c, d**) Double fluorescent ISH shows that the gene for Gαolf subunit of heterotrimeric G protein (green fluorescence), characteristic of OSNs, is co-expressed with *Olfr692* (red) in the MOE (**d**), but is not expressed in the VNO (absent green fluorescent signal in **c**). Nuclear staining is To-Pro-3 labeling (blue). Microscopy images are representative from the set of scored sections indicated in Additional file 10: Table S2. (**e**) Double fluorescent ISH shows that *Olfr692* (green) and *Olfr78* (red) are not co-expressed in the same cells, suggesting that their expression is singular (images representative from a set of 16 sections, from four mice). Quantification of co-labeling counts is summarized in Additional file 10: Table S2. lu, VNO lumen; ep, MOE sensory epithelium. Scale bars represent 100 μm. (PDF 2.18 MB) Additional file 4: Figure S3. Investigation of co-expression of *Olfr692* with genes for V2R vomeronasal receptors in clade A. (**a**–**d**) Double in situ hybridization on VNO sections showing that *Olfr692*-positive cells do not overlap with the expression of genes for V2R receptors in clade A, detected with probes able to recognize members of subclades A1 (**a**), A4 (**b**), A5 (**c** and **c’**), A8 (**d**), A9 (**e**), A2 (**f**) and A3 (**g**). Middle and right panels in (**a**–**d**) and left and right panels in (**e**–**g**) show the overlay between *Olfr692* staining (red fluorescence in **a–d** and chromogenic staining with BCIP/NBT in purple in **e**–**g**), To-Pro-3 nuclear staining (blue signal) and staining for V2R receptor genes (chromogenic development in **a**–**d** and fluorescent staining in **e**–**g**). For **a**, **b** and **d**–**g**, the right panel is a higher magnification image to evidence absence of co-expression. Microscopy images are representative from the set of scored sections indicated in Additional file 10: Table S2. Details on quantification of co-labeling counts are summarized in Additional file 10: Table S2. lu, VNO lumen. Scale bars represent 100 μm; panels without scale bars have the same magnification as the top leftmost panel. Nuclear staining is To-Pro-3 labeling (blue). (PDF 1.36 MB) Additional file 5: Figure S4. Investigation of co-expression of *Olfr692* with genes for V2R vomeronasal receptors in clades B, C and D, and H2-Mv MHC molecules. (**a, b**) Double in situ hybridization (ISH) on VNO sections showing that *Olfr692*-positive cells do not overlap with the expression of *V2R* genes in clades B (**a**) and D (**b**). Middle and right panels show the overlay between staining for *Olfr692* (red), To-Pro-3 (blue), and *V2Rs*. The right panels are higher magnification images of the middle panels to evidence absence of co-expression. (**c**) Control staining to show that the microscopy technique used is sufficient to visualize ISH fluorescent signal (left) even in the presence of overlapping purple precipitate from chromogenic development (middle and right panels). (**d, e**) Double fluorescent ISH to investigate co-expression of *Olfr692* (red) and *V2R* receptor genes in clade C (green). Insets are higher magnification images to evidence high co-expression with Vmn2r2 (**d**) but limited co-expression with Vmn2r1 (**e**). (**f**) Double fluorescent ISH experiment showing absence of co-localization of fluorescent signals for *Olfr692* (green) and a probe for H2-Mv member *M10.2* (red). (**f’**) Another example of absence of *M10.2* and *Olfr692* co-expression (higher magnification image in inset). (**g**) Chromogenic ISH with two probes for H2-Mv members *M10.5* and *M10.6* (middle panel) combined with fluorescent detection of *Olfr692* (red; left panel) reveals partial co-localization. The right panel is an overlay between the chromogenic signal, false-colored in white, and fluorescence, evidencing co-labeling (yellow arrowheads). Microscopy images are representative from the set of scored sections indicated in Additional file 10: Table S2. Details on quantification of co-labeling counts are summarized in Additional file 10: Table S2. lu, VNO lumen. Scale bars represent 100 μm; panels without scale bars have the same magnification as the top leftmost panel. Nuclear staining is To-Pro-3 labeling (blue). (PDF 1.54 MB) Additional file 6: Figure S5. In situ hybridization (ISH) probe validation for highly similar genes in the V2R family of vomeronasal receptors, expressed in the basal VNO layer. (**a**) Double fluorescent ISH with two probes labeled with different haptens (fluorescein, FLU, marked in green fluorescence, and digoxigenin, DIG, in red fluorescence) designed to detect the same *V2R* gene results in labeling of the same cells, showing that the ISH protocol and subsequent fluorescent detection consistently and robustly label the same subpopulation of VNO sensory neurons with probes for the same gene. (**b–d**) Double fluorescent ISH with probes for two genes in the same clade of V2R receptors (left panel) leads to labeling of largely overlapping subsets of cells in the VNO (middle panel) for receptors in clades A4 (**b** and **c**) and A1 (**d**). The right panels show quantification of singly (green and red leftmost bars) or doubly (yellow rightmost bar) stained cells per VNO section. (**e**) Probes for a pair of V2R receptors in the large A8 clade (*Vmn2r90* and *Vmn2r107*; left) do not result in significant co-labeling in double ISH experiments (middle and right panels). Therefore, in subsequent experiments, a combination of *Vmn2r90* and *Vmn2r107* probes was used for this clade. (**f, g**) Probes for receptors in different V2R subclades [A4 and A3 (**f**) and A4 and A8 (**g**)] do not result in significant co-labeling in double fluorescent ISH experiments. Images are representative from 16–24 sections, from 4-6 mice. For the cell counts in the right panels, calculations were performed over n = 12 sections, from four mice. lu, VNO lumen. Scale bars represent 100 μm. (PDF 627 kb) Additional file 7: Table S1. Quantification of activity in *Olfr692*-positive cells by double in situ hybridization. Quantification of labeled cells in experiments to investigate co-localization between *Olfr692* and the VNO neuronal activation marker *Egr1* in VNO sections from animals exposed to a range of odorous stimuli (left column). Each n indicates the number of animals studied. For most subjects, one or two randomly chosen sections were used per animal, and the cell count for each subject was the mean calculated from counts taken from those sections. The total number of imaged sections is indicated in the third column. Mean ± SEM. (DOCX 15 kb) Additional file 8: Figure S6. Investigation of activation of *Olfr692*-expressing cells by aliphatic acids in vivo and odorant screening in *Olfr692*-expressing heterologous cells using SEAP assay. (**a**) *Olfr692*-expressing cells are not activated by short-chain aliphatic acids, such as heptanoic, octanoic, nonanoic, and decanoic acids. See also Additional file 7: Table S1 and references [49, 50]. (**b**) Screening of *Olfr692*-expressing artificially modified Hana3a heterologous cells with aliphatic acids presented at different concentrations shows no stimulus-induced activity (alkaline phosphatase output in black lines; positive control response for receptor Olfr544 [49] in red line). Responses were normalized against maximum response in Olfr544-expressing control cells exposed to nonanedioic acid. Mean ± SEM. lu, VNO lumen. Scale bars represent 100 μm. Nuclear staining is To-Pro-3 labeling (blue). (**c**) Functional assay with a panel of 70 odorants in *Olfr692*-expressing Hana3a cells. Although serial dilutions in half-log steps from 10^–3^–10^–7^ M were used, only the concentrations which gave the maximum fold-change for Olfr692 are shown for each odorant. The x-axis indicates the SEAP signal fold-change for *Olfr692*-expressing cells relative to Hana3a cells with no OR. The y-axis shows signal fold-change over baseline. Stimulus categories are color-coded, to indicate stimuli that activate VSNs in vitro, pheromones and kairomones, stimuli that can still be detected by mice even in the absence of a functional MOE (AC3-/- mice), or known class I OR ligands. (**c’**) Immunocytochemistry against the Rho tag to show cell surface expression of Olfr692 (red) in Hana3a transfected cells. (**d**) Full concentration-response curves for odorants which gave a fold-change over baseline ≥2.5 and fold-change over no-OR control >1.0 in (**c**). P values are for interaction of receptor and concentration of odorant, two-way ANOVA. Colored lines indicate Hana3a cells expressing Olfr692, in comparison with "no-OR" control cells (black lines). (PDF 1.97 MB) Additional file 9: Figure S7. Supporting experiments on activation of the *Olfr692*-expressing population by pup odors. (**a**) Double in situ hybridization (ISH) experiment with probes for *Egr1* (red fluorescence) and *Olfr692* (green) to show that pup odors collected by washing pups with warm PBS and deposited on gauze are able to activate the *Olfr692*-expressing population in vivo. Images are representative from a set of 20 imaged sections, from n = 4 animals. (**b**) *Egr1/Olfr692* double ISH experiment to show that pups placed inside a plastic capsule bearing 4-mm wide holes are able to activate the *Olfr692*-expressing population. Images are representative from a set of 20 imaged sections, from n = 4 animals. Mean ± SEM. lu, VNO lumen. Scale bars represent 25 μm. Nuclear staining is To-Pro-3 labeling (blue). (PDF 118 kb) Additional file 10: Table S2. Quantification of co-labeling in double in situ hybridization (ISH) experiments with cell type-specific molecular markers. Numbers of singly or doubly labeled cells in double ISH experiments to investigate co-localization between *Olfr692* and several molecular markers of VNO and MOE neurons. Each n indicates the number of sections studied. The total number of mice from which these sections were taken is indicated in the thirds column. Mean ± SEM; n.d. = non-determined. (DOCX 15 kb)

## Abbreviations

CNGA: Cyclic-nucleotide gated channel
FPKM: Fragments per kilobase of exon sequence per million fragments
GPCR: G-protein-coupled receptor
MOE: Main olfactory epithelium
OR: Odorant receptor
OSN: Olfactory sensory neuron
TrpC2: Transient receptor potential family member C2 ion channel
VNO: Vomeronasal organ
VSN: Vomeronasal sensory neuron
