# Supplementary material for: Detection of pup odors by non-canonical adult vomeronasal neurons expressing an odorant receptor gene is influenced by sex and parenting status
Source: BMC Biol. 2016 Feb 15;14:12. doi: 10.1186/s12915-016-0234-9 (PMC4753656; doi:10.1186/s12915-016-0234-9)
Supplement: Additional file 1: Figure S1. — Investigation of expression of OR genes in the VNO by in situ hybridization (ISH) and supporting control data for Olfr692 expression. (a) Olfr691, the OR gene located closest to Olfr692 in the mouse genome, is not expressed in the VNO, nor are two other OR genes not found in the VNO RNA sequencing library (Olfr638 and Olfr569). The same probes label several neurons in the MOE. Images are representative from sets of 12 sections, from 3 mice. (b-d) Chromogenic (left panels) and fluorescent (right) ISH images with probes for V2R receptor genes Vmn2r41 (b), Vmn2r107 (c) and Vmn2r69 (d). See also Additional file 2: Dataset S1 and Additional file 6: Figure S5 for probe validations. (e) Chromogenic (left) or fluorescent (right) ISH for Olfr78 in the VNO (representative from 14 sections, 7 mice). (f) Quantitation of location of Olfr692- and Olfr78-positive cells in the VNO apical (blue bars) or basal (red bars) zones (error bars are SEM; n = 48 sections, 4 sections per mouse, for Olfr692; n = 12 sections, 2 sections per mouse, for Olfr78). See also Additional file 3: Figure S2e. (g) Chromogenic ISH for Olfr1512 on VNO (left; representative from 27 sections, 3 mice) and MOE sections (right; representative from 24 sections). (h-j) Absence of VNO expression for Olfr124 (h, left and middle panels; representative from 32 sections, 16 mice) and Olfr1509 (i; representative from 8 sections, 4 mice). Probe validations in the MOE for Olfr124 (h, right; representative from 12 sections, 6 animals) and Olfr1509 (j; representative from 8 sections, 4 mice). (k) Olfr124 is highly expressed in the Septal Organ of Masera (representative from 12 sections, 4 mice). lu, VNO lumen; ep, MOE sensory epithelium; som, Septal Organ of Masera (SOM); spt, nasal septum. Scale bars represent 100 µm. Nuclear staining is DAPI labeling (blue). (PDF 1.56 MB) [file 12915_2016_234_MOESM1_ESM.pdf]

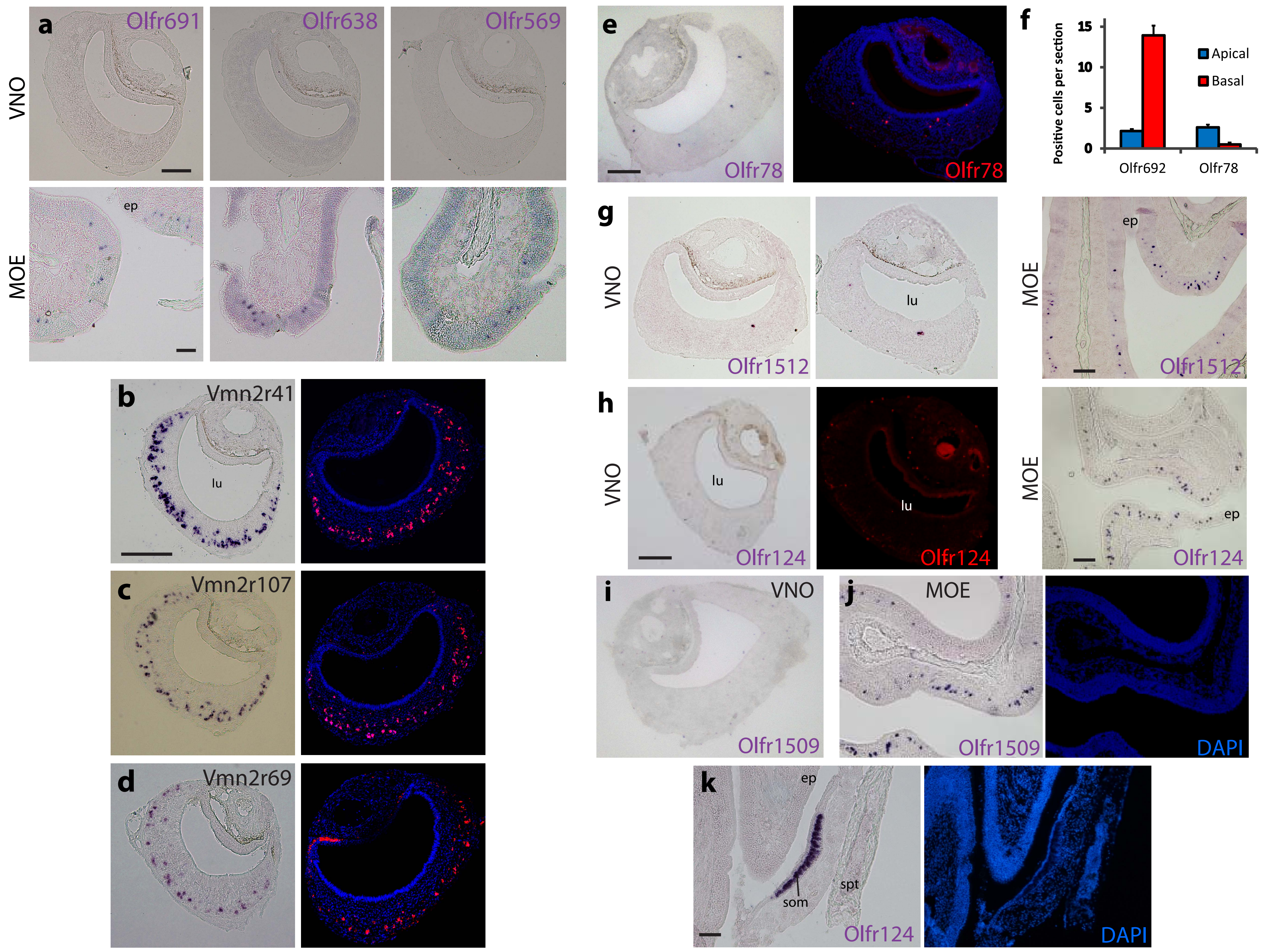

a
VNO
MOE
Olfr691
Olfr638
Olfr569
ep
b
Vmn2r41
lu
c
Vmn2r107
d
Vmn2r69
e
Olfr78
Olfr78
f
Positive cells per section
15
10
5
0
Apical
Basal
Olfr692
Olfr78
g
VNO
Olfr1512
lu
MOE
ep
Olfr1512
h
VNO
lu
Olfr124
Olfr124
MOE
ep
Olfr124
i
VNO
Olfr1509
j
MOE
Olfr1509
DAPI
k
ep
spt
som
Olfr124
DAPI

Additional discussion on Figure S1.

(b) When we used a probe based on the *Vmn2r41* gene, which cross-hybridizes to 23 members in the A4 clade of V2R receptor genes with which it shares more than 90% similarity at the nucleotide level (Additional file 2: Dataset S1), we found 98.1 ± 5.0 stained cells per section (mean ± s.e.m.; n=17 sections, from 8 mice), meaning that there is an average of 4-5 VNO cells per section expressing each receptor gene in clade A4, which is less than the number of *Olfr692*-positive cells at the same age.

(c) When we used a probe based on the *Vmn2r107* gene, which cross-hybridizes to 15 members in the A8 clade of V2R receptor genes with which it shares more than 90% similarity at the nucleotide level (Additional file 2: Dataset S1), we found 101.2 ± 6.6 stained cells per section (mean ± s.e.m.; n=12 sections, from 6 mice), indicating an average of 6-7 stained cells per section per receptor gene in this clade.

(d) With a probe based on the *Vmn2r69* gene, which recognizes 10 receptor genes in the V2R A5 clade with which it shares more than 85% similarity at the nucleotide level, we found 39.1 ± 3.4 stained cells per section (mean ± s.e.m.; n=15 sections, from 8 mice), indicating an average of 3-4 cells per section expressing each receptor gene in this clade.
