# Supplementary figures and images for: Detection of pup odors by non-canonical adult vomeronasal neurons expressing an odorant receptor gene is influenced by sex and parenting status
Source: BMC Biol. 2016 Feb 15;14:12. doi: 10.1186/s12915-016-0234-9 (PMC4753656; doi:10.1186/s12915-016-0234-9)

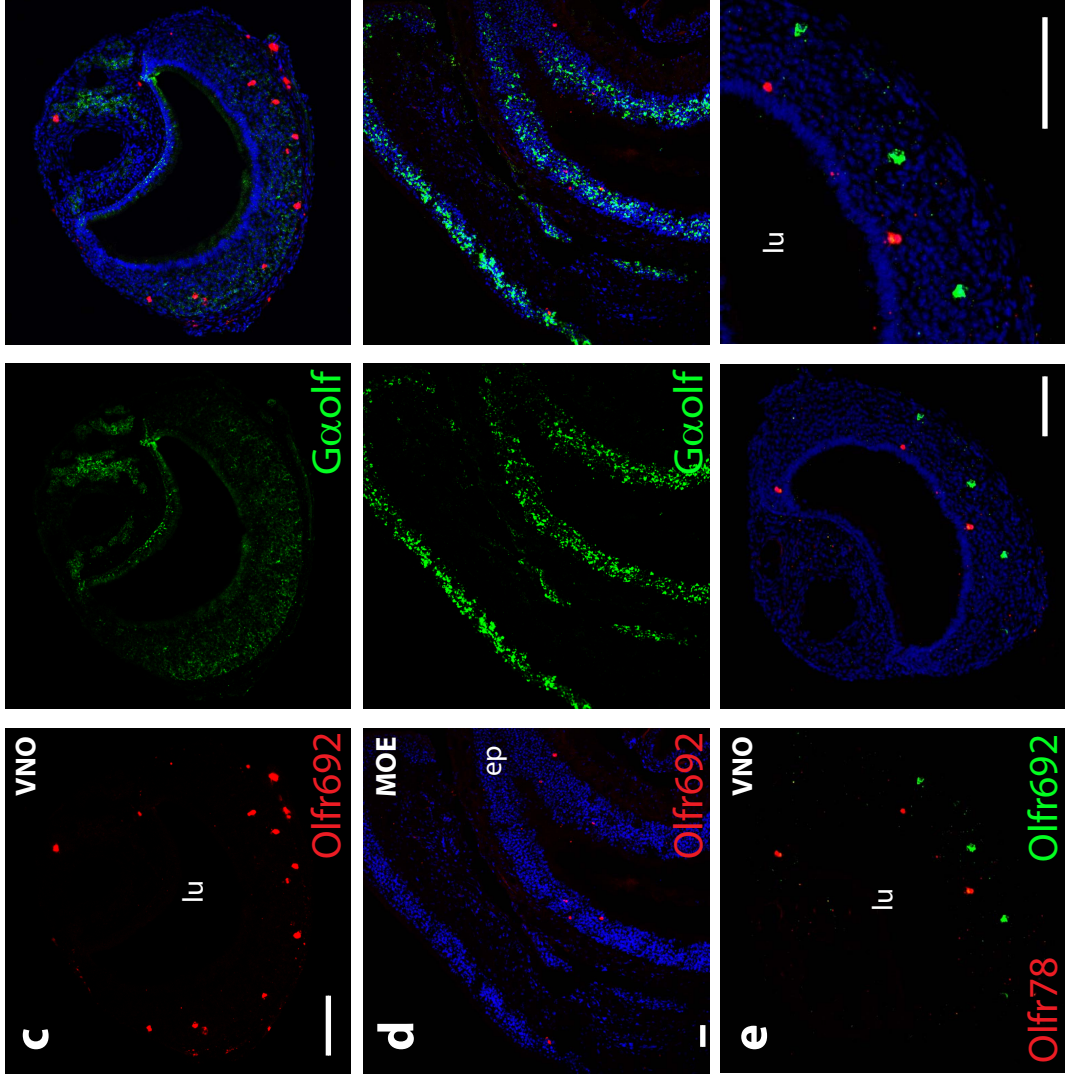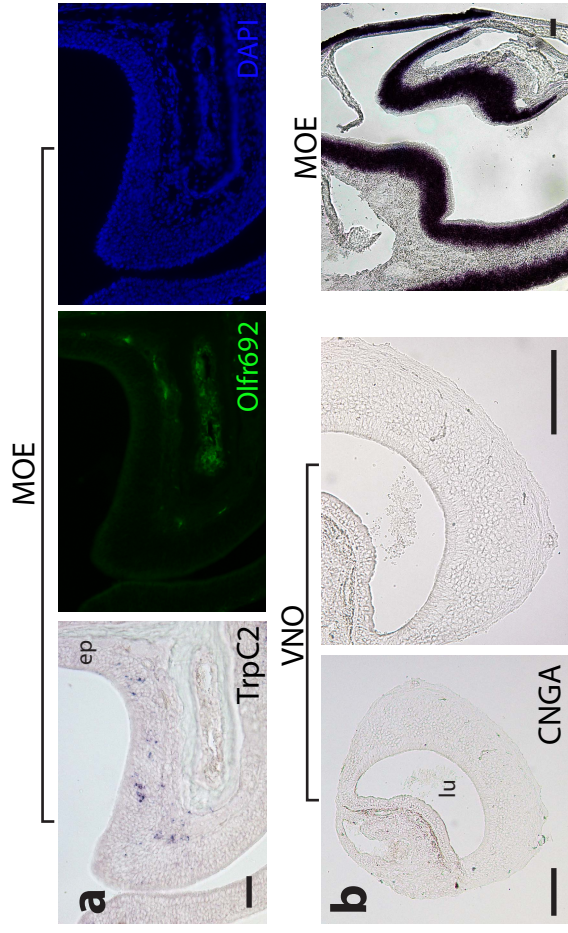

Supplement: Additional file 3: Figure S2. — Control experiments for in situ hybridization investigation (ISH) of the expression of genes for signal transduction molecules in Olfr692-positive cells. (a) The gene for transient receptor potential member TrpC2, characteristic of VSNs, is expressed in a very limited subset of MOE sensory neurons (chromogenic detection following ISH), but these OSNs do not express Olfr692, which would appear as green fluorescent cells in the middle panel (images representative from a set of 21 sections, from seven mice). Blue is DAPI nuclear staining. (b) The gene for cyclic nucleotide gated channel subunit cyclic-nucleotide gated channel (CNGA2), characteristic of MOE OSNs (chromogenic ISH in the right panel), is not expressed in the VNO (left). Middle panel shows a higher magnification image of the leftmost panel. For the VNO, images are representative from a set of 12 sections, from six mice; for the MOE, images are from a set of 12 sections, from three mice. (c, d) Double fluorescent ISH shows that the gene for Gαolf subunit of heterotrimeric G protein (green fluorescence), characteristic of OSNs, is co-expressed with Olfr692 (red) in the MOE (d), but is not expressed in the VNO (absent green fluorescent signal in c). Nuclear staining is To-Pro-3 labeling (blue). Microscopy images are representative from the set of scored sections indicated in Additional file 10: Table S2. (e) Double fluorescent ISH shows that Olfr692 (green) and Olfr78 (red) are not co-expressed in the same cells, suggesting that their expression is singular (images representative from a set of 16 sections, from four mice). Quantification of co-labeling counts is summarized in Additional file 10: Table S2. lu, VNO lumen; ep, MOE sensory epithelium. Scale bars represent 100 μm. (PDF 2.18 MB) [file 12915_2016_234_MOESM3_ESM.pdf]

## V2R clade A

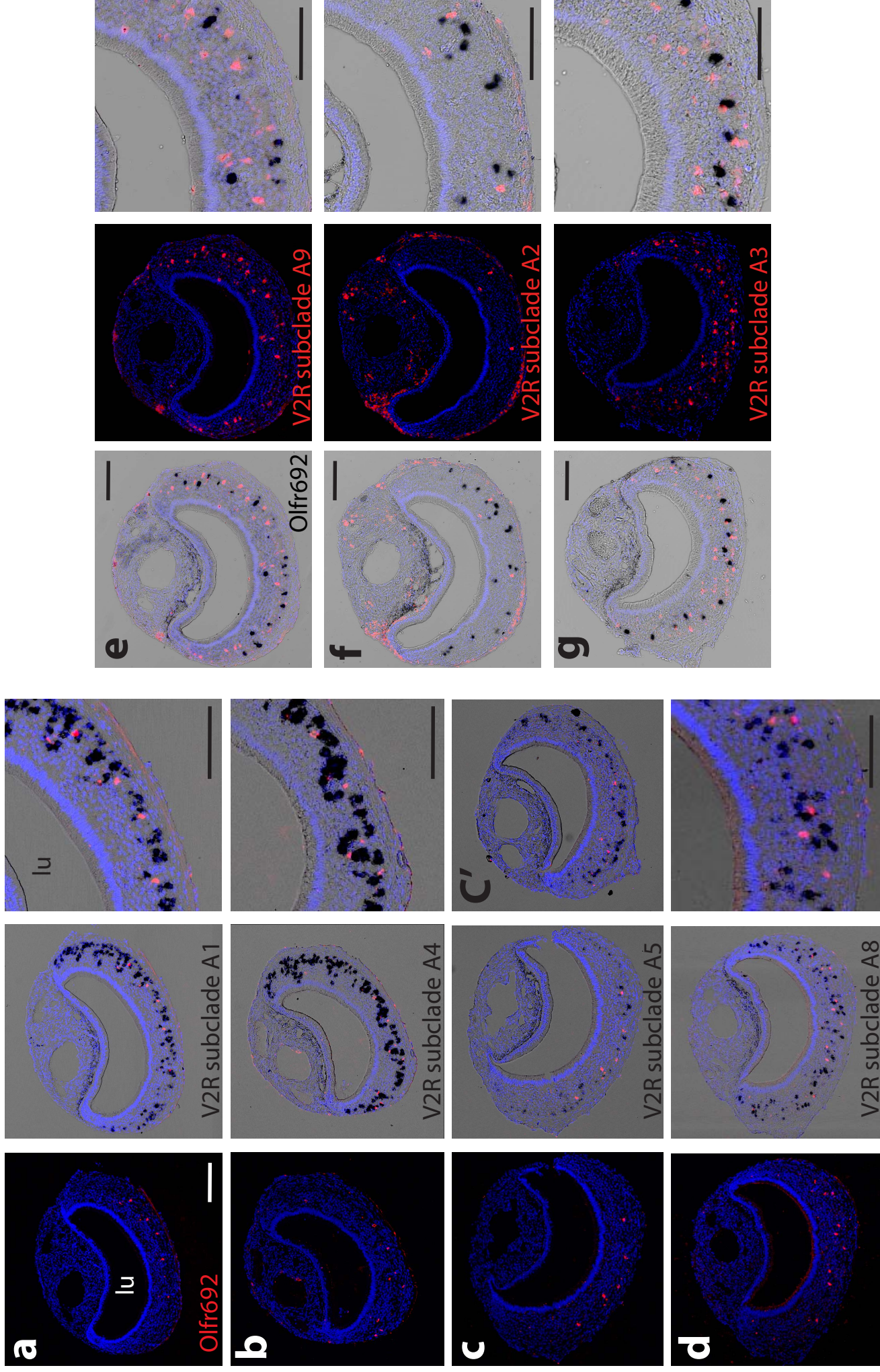

Supplement: Additional file 4: Figure S3. — Investigation of co-expression of Olfr692 with genes for V2R vomeronasal receptors in clade A. (a–d) Double in situ hybridization on VNO sections showing that Olfr692-positive cells do not overlap with the expression of genes for V2R receptors in clade A, detected with probes able to recognize members of subclades A1 (a), A4 (b), A5 (c and c’), A8 (d), A9 (e), A2 (f) and A3 (g). Middle and right panels in (a–d) and left and right panels in (e–g) show the overlay between Olfr692 staining (red fluorescence in a–d and chromogenic staining with BCIP/NBT in purple in e–g), To-Pro-3 nuclear staining (blue signal) and staining for V2R receptor genes (chromogenic development in a–d and fluorescent staining in e–g). For a, b and d–g, the right panel is a higher magnification image to evidence absence of co-expression. Microscopy images are representative from the set of scored sections indicated in Additional file 10: Table S2. Details on quantification of co-labeling counts are summarized in Additional file 10: Table S2. lu, VNO lumen. Scale bars represent 100 μm; panels without scale bars have the same magnification as the top leftmost panel. Nuclear staining is To-Pro-3 labeling (blue). (PDF 1.36 MB) [file 12915_2016_234_MOESM4_ESM.pdf]

V2R clade B

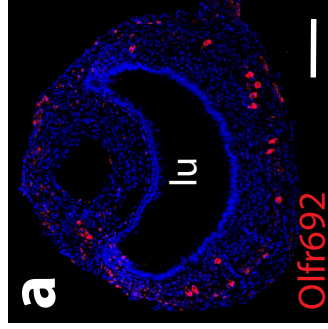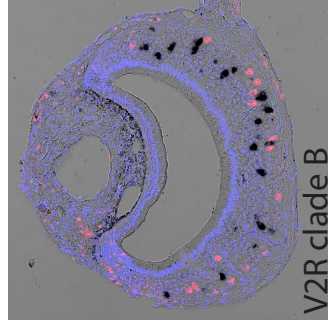

V2R clade D

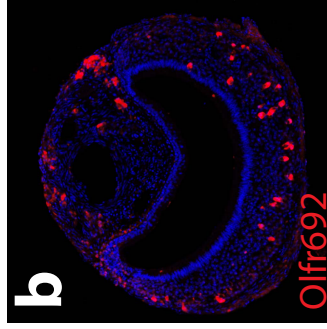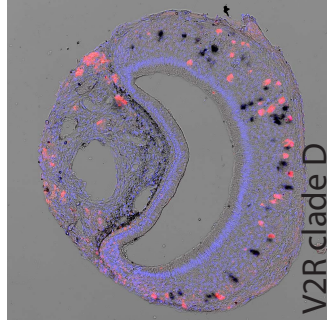

V2R clade C

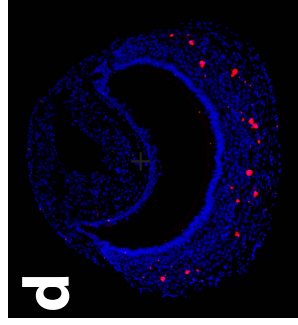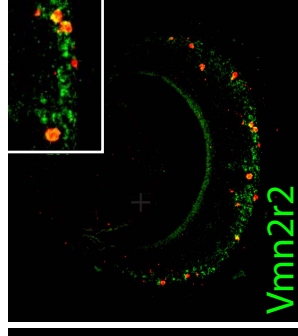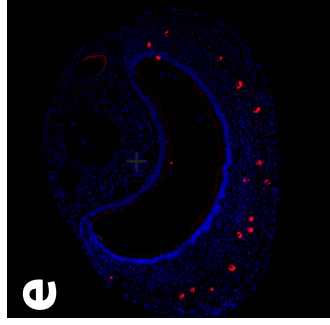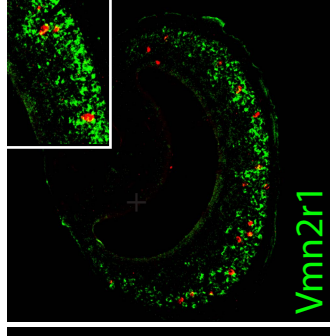

H2-Mv family

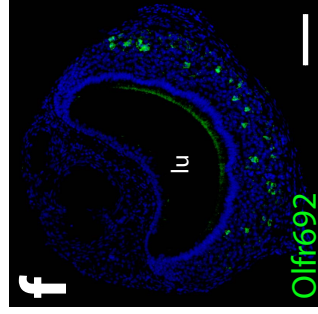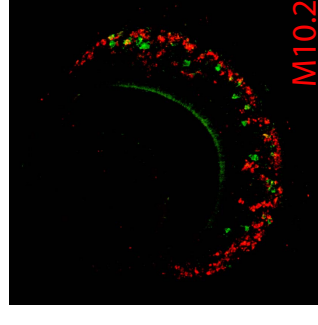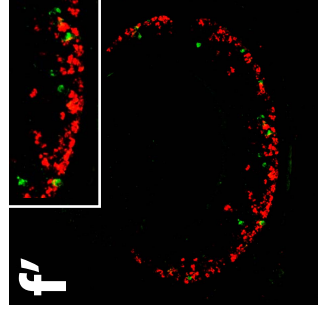

Co-labeling control

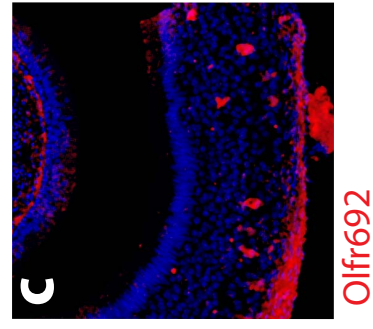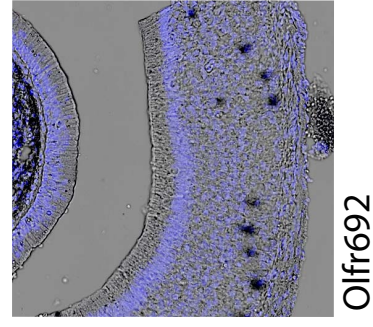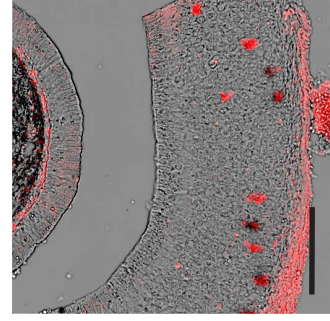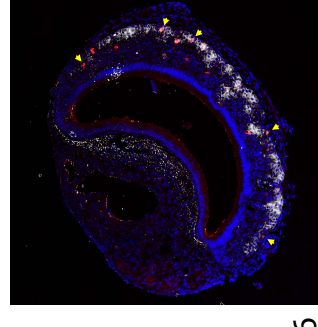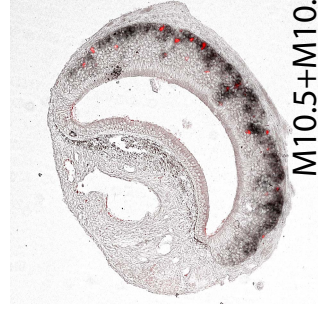

M10.5+M10.6

Supplement: Additional file 5: Figure S4. — Investigation of co-expression of Olfr692 with genes for V2R vomeronasal receptors in clades B, C and D, and H2-Mv MHC molecules. (a, b) Double in situ hybridization (ISH) on VNO sections showing that Olfr692-positive cells do not overlap with the expression of V2R genes in clades B (a) and D (b). Middle and right panels show the overlay between staining for Olfr692 (red), To-Pro-3 (blue), and V2Rs. The right panels are higher magnification images of the middle panels to evidence absence of co-expression. (c) Control staining to show that the microscopy technique used is sufficient to visualize ISH fluorescent signal (left) even in the presence of overlapping purple precipitate from chromogenic development (middle and right panels). (d, e) Double fluorescent ISH to investigate co-expression of Olfr692 (red) and V2R receptor genes in clade C (green). Insets are higher magnification images to evidence high co-expression with Vmn2r2 (d) but limited co-expression with Vmn2r1 (e). (f) Double fluorescent ISH experiment showing absence of co-localization of fluorescent signals for Olfr692 (green) and a probe for H2-Mv member M10.2 (red). (f’) Another example of absence of M10.2 and Olfr692 co-expression (higher magnification image in inset). (g) Chromogenic ISH with two probes for H2-Mv members M10.5 and M10.6 (middle panel) combined with fluorescent detection of Olfr692 (red; left panel) reveals partial co-localization. The right panel is an overlay between the chromogenic signal, false-colored in white, and fluorescence, evidencing co-labeling (yellow arrowheads). Microscopy images are representative from the set of scored sections indicated in Additional file 10: Table S2. Details on quantification of co-labeling counts are summarized in Additional file 10: Table S2. lu, VNO lumen. Scale bars represent 100 μm; panels without scale bars have the same magnification as the top leftmost panel. Nuclear staining is To-Pro-3 labeling (blue). (PDF 1.54 MB) [file 12915_2016_234_MOESM5_ESM.pdf]

**a**

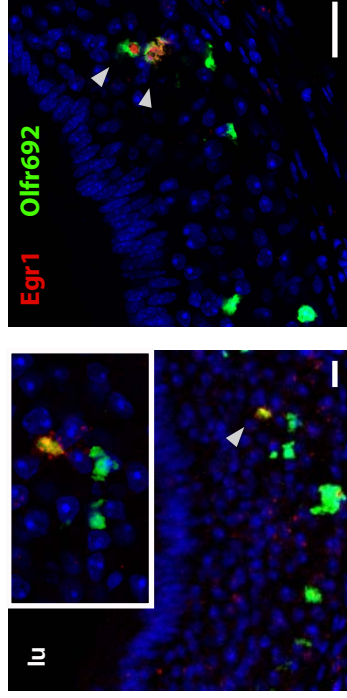

**b**

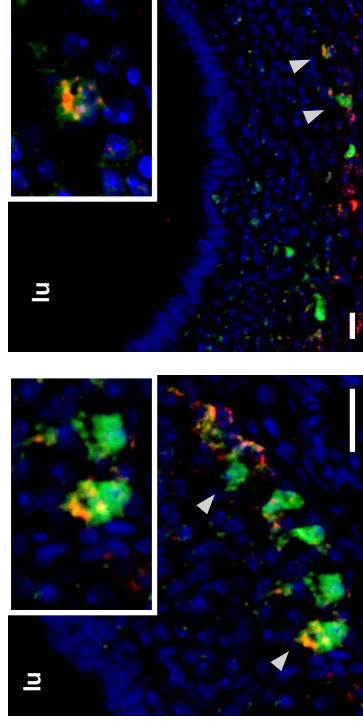

Supplement: Additional file 9: Figure S7. — Supporting experiments on activation of the Olfr692-expressing population by pup odors. (a) Double in situ hybridization (ISH) experiment with probes for Egr1 (red fluorescence) and Olfr692 (green) to show that pup odors collected by washing pups with warm PBS and deposited on gauze are able to activate the Olfr692-expressing population in vivo. Images are representative from a set of 20 imaged sections, from n = 4 animals. (b) Egr1/Olfr692 double ISH experiment to show that pups placed inside a plastic capsule bearing 4-mm wide holes are able to activate the Olfr692-expressing population. Images are representative from a set of 20 imaged sections, from n = 4 animals. Mean ± SEM. lu, VNO lumen. Scale bars represent 25 μm. Nuclear staining is To-Pro-3 labeling (blue). (PDF 118 kb) [file 12915_2016_234_MOESM9_ESM.pdf]
