# Supplementary material for: Detection of pup odors by non-canonical adult vomeronasal neurons expressing an odorant receptor gene is influenced by sex and parenting status
Source: BMC Biol. 2016 Feb 15;14:12. doi: 10.1186/s12915-016-0234-9 (PMC4753656; doi:10.1186/s12915-016-0234-9)
Supplement: Additional file 6: Figure S5. — In situ hybridization (ISH) probe validation for highly similar genes in the V2R family of vomeronasal receptors, expressed in the basal VNO layer. (a) Double fluorescent ISH with two probes labeled with different haptens (fluorescein, FLU, marked in green fluorescence, and digoxigenin, DIG, in red fluorescence) designed to detect the same V2R gene results in labeling of the same cells, showing that the ISH protocol and subsequent fluorescent detection consistently and robustly label the same subpopulation of VNO sensory neurons with probes for the same gene. (b–d) Double fluorescent ISH with probes for two genes in the same clade of V2R receptors (left panel) leads to labeling of largely overlapping subsets of cells in the VNO (middle panel) for receptors in clades A4 (b and c) and A1 (d). The right panels show quantification of singly (green and red leftmost bars) or doubly (yellow rightmost bar) stained cells per VNO section. (e) Probes for a pair of V2R receptors in the large A8 clade (Vmn2r90 and Vmn2r107; left) do not result in significant co-labeling in double ISH experiments (middle and right panels). Therefore, in subsequent experiments, a combination of Vmn2r90 and Vmn2r107 probes was used for this clade. (f, g) Probes for receptors in different V2R subclades [A4 and A3 (f) and A4 and A8 (g)] do not result in significant co-labeling in double fluorescent ISH experiments. Images are representative from 16–24 sections, from 4-6 mice. For the cell counts in the right panels, calculations were performed over n = 12 sections, from four mice. lu, VNO lumen. Scale bars represent 100 μm. (PDF 627 kb) [file 12915_2016_234_MOESM6_ESM.pdf]

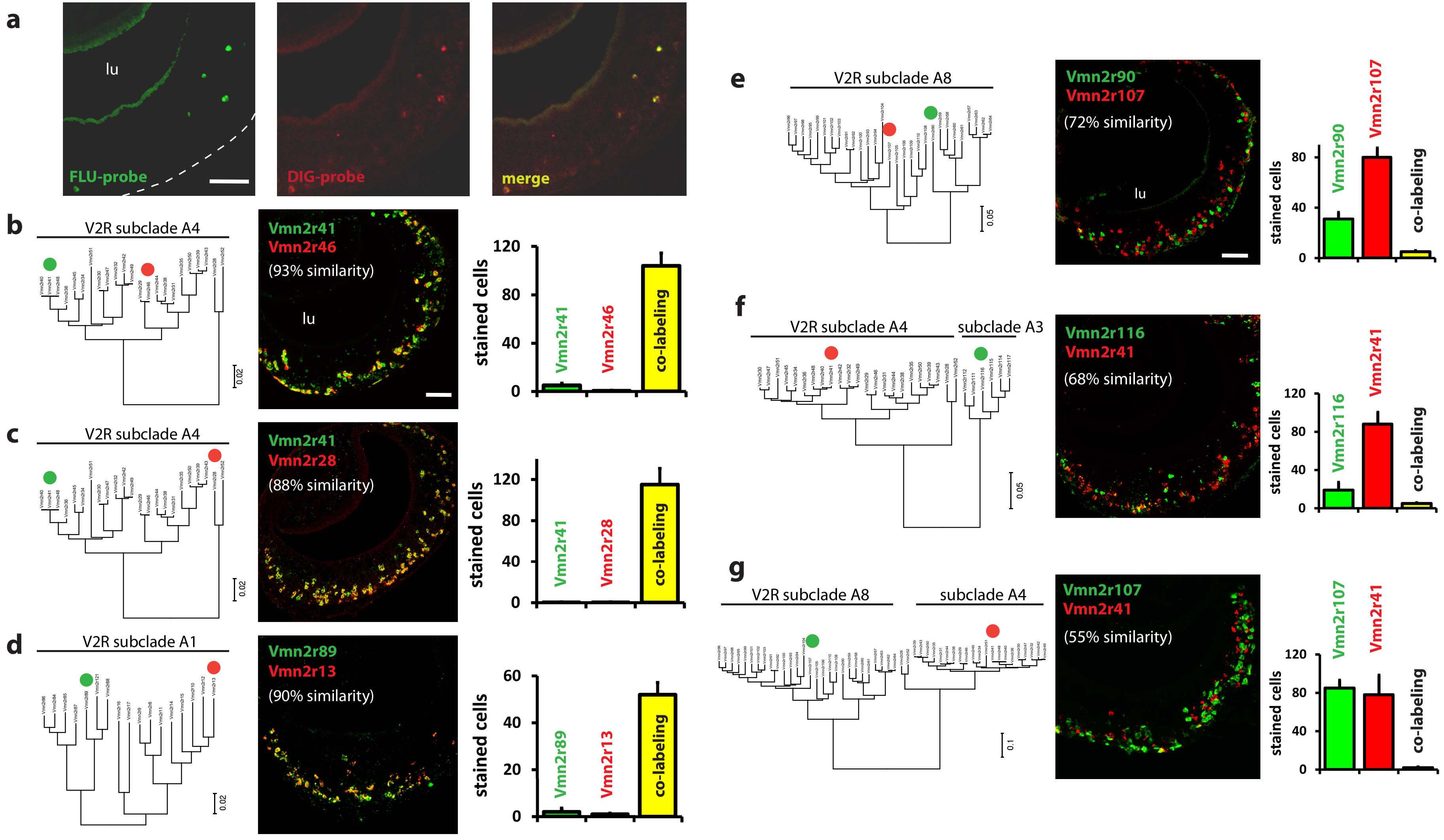

a
lu
FLU-probe
DIG-probe
merge
b
V2R subclade A4
0.02
Vmn2r41
Vmn2r46
(93% similarity)
lu
stained cells
120
80
40
0
Vmn2r41
Vmn2r46
co-labeling
c
V2R subclade A4
0.02
Vmn2r41
Vmn2r28
(88% similarity)
stained cells
120
80
40
0
Vmn2r41
Vmn2r28
co-labeling
d
V2R subclade A1
0.02
Vmn2r89
Vmn2r13
(90% similarity)
stained cells
60
40
20
0
Vmn2r89
Vmn2r13
co-labeling
e
V2R subclade A8
0.05
Vmn2r90
Vmn2r107
(72% similarity)
lu
stained cells
80
40
0
Vmn2r90
Vmn2r107
co-labeling
f
V2R subclade A4
subclade A3
0.05
Vmn2r116
Vmn2r41
(68% similarity)
stained cells
120
80
40
0
Vmn2r116
Vmn2r41
co-labeling
g
V2R subclade A8
subclade A4
0.1
Vmn2r107
Vmn2r41
(55% similarity)
stained cells
120
80
40
0
Vmn2r107
Vmn2r41
co-labeling

Additional discussion on Figure S5.

The phylogenetic trees in the left panels were produced using the maximum-likelihood method with the Kimura-Nei matrix, and vertical scale bars indicate genetic distances. The middle panels are representative double fluorescent ISH images, where one probe results in green fluorescent labeling and the other leads to red fluorescence. The similarity between the two tested genes in the probe region is shown in the middle panel (see also Additional file 2: Dataset S1 for the complete pairwise comparisons between V2R receptors).

(b-d) For clades where receptors share more than 75 % similarity, exemplified here by clades A4 and A1, results show that probes for two different genes in the same clade recognize the same subset of cells. Therefore, we decided to use one or two probes in each clade to label sensory neurons expressing receptors in that clade in subsequent experiments to characterize the *Olfr692*-expressing population in the VNO (except for clade A8; see panel **e**).

(e) For clade A8, which is the largest in the V2R family, possessing two subgroups of receptors that share less than 75 % sequence similarity (left panel; see also Additional file 2: Dataset S1), we tested probes for different receptor genes. Because overlapping staining for these probes was limited, we used a combination of two probes (for *Vmn2r107* and *Vmn2r90* genes) in subsequent experiments.

(f, g) We tested probes for receptors in distinct V2R subclades, which share less than 75 % sequence similarity (see Additional file 2: Dataset S1). The results indicate that a probe in one clade does not cross-hybridize to other clades of V2R receptors and can therefore be used to label the target clade with confidence.
