# Supplementary material for: Detection of pup odors by non-canonical adult vomeronasal neurons expressing an odorant receptor gene is influenced by sex and parenting status
Source: BMC Biol. 2016 Feb 15;14:12. doi: 10.1186/s12915-016-0234-9 (PMC4753656; doi:10.1186/s12915-016-0234-9)
Supplement: Additional file 7: Table S1. — Quantification of activity in Olfr692-positive cells by double in situ hybridization. Quantification of labeled cells in experiments to investigate co-localization between Olfr692 and the VNO neuronal activation marker Egr1 in VNO sections from animals exposed to a range of odorous stimuli (left column). Each n indicates the number of animals studied. For most subjects, one or two randomly chosen sections were used per animal, and the cell count for each subject was the mean calculated from counts taken from those sections. The total number of imaged sections is indicated in the third column. Mean ± SEM. (DOCX 15 kb) [file 12915_2016_234_MOESM7_ESM.docx]

**Table S1. Quantification of activity in *Olfr692*-positive cells by double *in situ* hybridization.**

| **Stimuli** | **number of mice**  **(n)** | **number of sections** | **total number of counted *Olfr692*-positive cells** | **% of all counted *Olfr692*-positive cells that also stain positive for *Egr1*** | | **% of *Olfr692*-positive cells per section that also stain positive for *Egr1*** |
| --- | --- | --- | --- | --- | --- | --- |
| Cat | 7 | 7 | 139 | | 2.1% | 2.1 ± 1.4% |
| Snake | 5 | 5 | 45 | | 3.7% | 3.2 ± 2.0% |
| Hawk | 4 | 4 | 33 | | 0 | 0 |
| Decanoic Acid 100mM | 5 | 5 | 93 | | 0 | 0 |
| Stearic Acid 100mM | 4 | 4 | 113 | | 0 | 0 |
| ♂ exp.♀ | 5 | 5 | 71 | | 0 | 0 |
| ♀ exp. ♂ | 5 | 5 | 69 | | 0 | 0 |
| ♀ exp. ♀ | 7 | 7 | 101 | | 4.0% | 5.0 ± 3.0% |
| ♂ exp. ♂ | 6 | 6 | 69 | | 2.9% | 3.0 ± 3.0% |
| Male adult exposed to milk | 6 | 12 | 206 | | 0 | 0 |
| ♂ exp. C3H juvenile ♀ (P15-P20) + ESP22 | 7 | 7 | 96 | | 0 | 0 |
| ♂ exp. C3H juvenile ♀ (P15-P20) + MPB | 6 | 6 | 147 | | 0 | 0 |
| ♂ exp. C57BL/6 juvenile ♀ (P15-P20) | 6 | 6 | 112 | | 23.2% | 23.7 ± 4.0% |
| Virgin ♂ exp. C57BL/6 juvenile (P0.5-P8.5) | 20 | 40 | 593 | | 23.8% | 27.4 ± 4.0% |
| Fathers (♂) exp. C57BL/6 juvenile ( P0.5-P8.5) | 6 | 48 | 634 | | 3.0% | 3.1 ± 0.8% |
| Virgin ♀ exp. C57BL/6 juvenile ( P0.5-P8.5) | 6 | 19 | 279 | | 3.2% | 3.4 ± 1.4% |
| Mothers (♀) exp. C57BL/6 juvenile ( P0.5-P8.5) | 4 | 28 | 614 | | 2.6% | 2.5 ± 0.8% |
| Virgin ♂ exp. to control odor | 14 | 14 | 181 | | 0 | 0 |
| ♂ TrpC2-/- exp. C57BL/6 juvenile ( P0.5-P8.5) | 9 | 9 | 15 | | 0 | 0 |
