# Supplementary material for: Detection of pup odors by non-canonical adult vomeronasal neurons expressing an odorant receptor gene is influenced by sex and parenting status
Source: BMC Biol. 2016 Feb 15;14:12. doi: 10.1186/s12915-016-0234-9 (PMC4753656; doi:10.1186/s12915-016-0234-9)
Supplement: Additional file 8: Figure S6. — Investigation of activation of Olfr692-expressing cells by aliphatic acids in vivo and odorant screening in Olfr692-expressing heterologous cells using SEAP assay. (a) Olfr692-expressing cells are not activated by short-chain aliphatic acids, such as heptanoic, octanoic, nonanoic, and decanoic acids. See also Additional file 7: Table S1 and references [49, 50]. (b) Screening of Olfr692-expressing artificially modified Hana3a heterologous cells with aliphatic acids presented at different concentrations shows no stimulus-induced activity (alkaline phosphatase output in black lines; positive control response for receptor Olfr544 [49] in red line). Responses were normalized against maximum response in Olfr544-expressing control cells exposed to nonanedioic acid. Mean ± SEM. lu, VNO lumen. Scale bars represent 100 μm. Nuclear staining is To-Pro-3 labeling (blue). (c) Functional assay with a panel of 70 odorants in Olfr692-expressing Hana3a cells. Although serial dilutions in half-log steps from 10–3–10–7 M were used, only the concentrations which gave the maximum fold-change for Olfr692 are shown for each odorant. The x-axis indicates the SEAP signal fold-change for Olfr692-expressing cells relative to Hana3a cells with no OR. The y-axis shows signal fold-change over baseline. Stimulus categories are color-coded, to indicate stimuli that activate VSNs in vitro, pheromones and kairomones, stimuli that can still be detected by mice even in the absence of a functional MOE (AC3-/- mice), or known class I OR ligands. (c’) Immunocytochemistry against the Rho tag to show cell surface expression of Olfr692 (red) in Hana3a transfected cells. (d) Full concentration-response curves for odorants which gave a fold-change over baseline ≥2.5 and fold-change over no-OR control >1.0 in (c). P values are for interaction of receptor and concentration of odorant, two-way ANOVA. Colored lines indicate Hana3a cells expressing Olfr692, in comparison with "no-OR" control cells (black lines). (P [file 12915_2016_234_MOESM8_ESM.pdf]

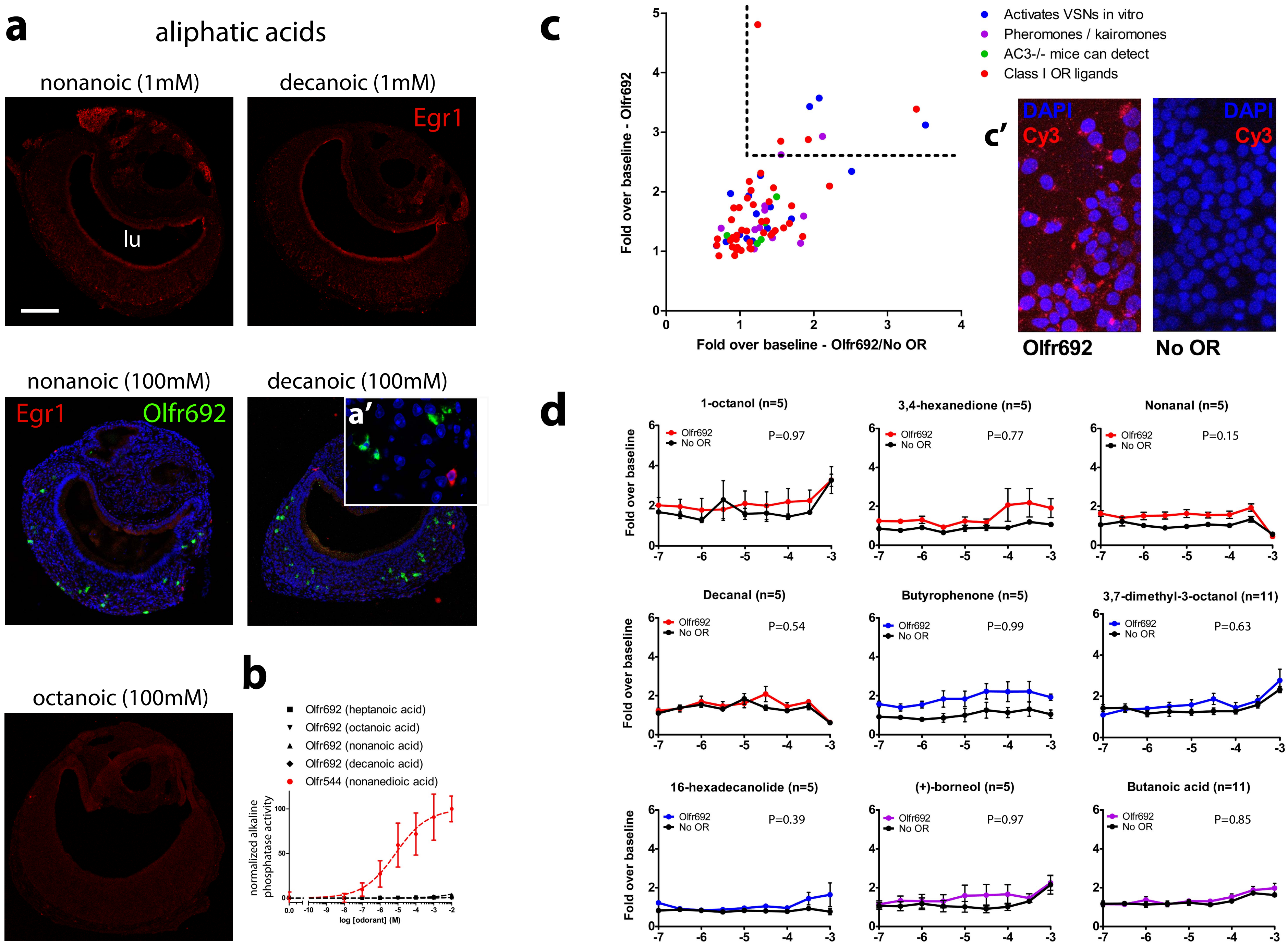
a
aliphatic acids
nonanoic (1mM)
decanoic (1mM)
Egr1
lu
nonanoic (100mM)
decanoic (100mM)
Egr1
Olfr692
a′
octanoic (100mM)
b
Olfr692 (heptanoic acid)
Olfr692 (octanoic acid)
Olfr692 (nonanoic acid)
Olfr692 (decanoic acid)
Olfr544 (nonanedioic acid)
normalized alkaline phosphatase activity
log [odorant] (M)
c
Activates VSNs in vitro
Pheromones / kairomones
AC3-/- mice can detect
Class I OR ligands
Fold over baseline - Olfr692
Fold over baseline - Olfr692/No OR
c′
DAPI
Cy3
Olfr692
No OR
d
1-octanol (n=5)
P=0.97
3,4-hexanedione (n=5)
P=0.77
Nonanal (n=5)
P=0.15
Decanal (n=5)
P=0.54
Butyrophenone (n=5)
P=0.99
3,7-dimethyl-3-octanol (n=11)
P=0.63
16-hexadecanolide (n=5)
P=0.39
(+)-borneol (n=5)
P=0.97
Butanoic acid (n=11)
P=0.85
Olfr692
No OR
Fold over baseline
log (M)

Additional discussion on Figure S6.

Class I ORs related to Olfr692 [2] have been shown to respond to short-chain aliphatic acids in heterologous cell assays [33, 49, 50]. However, in situ hybridization (ISH) for *Egr1* in **(a)** shows that nonanoic (C9) and decanoic (C10) acids presented to male C57BL/6 mice at 1 mM concentration (in mineral oil) do not activate the VNO sensory epithelium (absence of red fluorescence in the top row). On the other hand, when presented at 100 mM concentration to male C57BL/6 mice, these compounds result in a limited number of activated cells (middle row), but these do not overlap with the expression of *Olfr692* (green fluorescence) in double fluorescent ISH experiments (higher magnification detail in **a'**). Octanoic (C8) acid does not activate the VNO at 100 mM (bottom row), nor does stearic acid (C18) (data not shown), similar to control *Egr1* staining in animals exposed to control odor (mineral oil).
