# Supplementary material for: Detection of pup odors by non-canonical adult vomeronasal neurons expressing an odorant receptor gene is influenced by sex and parenting status
Source: BMC Biol. 2016 Feb 15;14:12. doi: 10.1186/s12915-016-0234-9 (PMC4753656; doi:10.1186/s12915-016-0234-9)
Supplement: Additional file 10: Table S2. — Quantification of co-labeling in double in situ hybridization (ISH) experiments with cell type-specific molecular markers. Numbers of singly or doubly labeled cells in double ISH experiments to investigate co-localization between Olfr692 and several molecular markers of VNO and MOE neurons. Each n indicates the number of sections studied. The total number of mice from which these sections were taken is indicated in the thirds column. Mean ± SEM; n.d. = non-determined. (DOCX 15 kb) [file 12915_2016_234_MOESM10_ESM.docx]

| **Gene X** | **total number of scored sections (n)** | **number of mice** | **total number of counted *Olfr692*-positive cells** | **% of all counted *Olfr692*-positive cells that also stain positive for gene X** | **% of *Olfr692*-positive cells per section that also stain positive for gene X** |
| --- | --- | --- | --- | --- | --- |
| *Trpc2* | 6 | 4 | 126 | 100.0% | 100.0 ± 0.0 % |
| *Gαo* | 6 | 6 | 90 | 100.0% | 100.0 ± 0.0 % |
| *Gαi2* | 8 | 3 | 112 | 6.3% | 5.0 ± 1.5 % |
| *Gαolf* | 10 | 4 | nd |  | no Gαolf expression in the VNO |
| *M10.2* | 6 | 4 | 88 | 9.1% | 9.2 ± 3.8 % |
| *M10.5/M10.6* | 6 | 4 | 70 | 84.3% | 83.1 ± 4.0 % |
| V2R Clade C (*Vmn2r1*) | 5 | 3 | 65 | 46.1% | 42.5 ± 8.0% |
| V2R Clade C (*Vmn2r2*) | 7 | 4 | 53 | 43.4% | 47.5 ± 4.2 % |
| V2R Clade A1 (*Vmn2r13+Vmn2r89*) | 11 | 4 | 119 | 5.0% | 5.1 ± 2.2% |
| V2R Clade A2 (*Vmn2r118*) | 5 | 3 | 90 | 0.0% | 0.0% |
| V2R Clade A3 (*Vmn2r116*) | 6 | 3 | 94 | 4.3% | 4.3 ± 1.5 % |
| V2R Clade A4 (*Vmn2r41+Vmn2r28*) | 7 | 4 | 101 | 6.0% | 6.3 ± 1.2 % |
| V2R Clade A5 (*Vmn2r69*) | 6 | 4 | 52 | 3.8% | 3.2 ± 2.1% |
| V2R Clade A8 (*Vmn2r107+Vmn2r90+Vmn2r58*) | 7 | 4 | 101 | 4.0% | 3.8 ± 1.4 % |
| V2R Clade A9 (*Vmn2r83*) | 5 | 3 | 79 | 1.3% | 1.5 ± 1.5 % |
| V2R Clade B (*Vmn2r20*) | 5 | 3 | 75 | 5.3% | 5.4 ± 1.4 % |
| V2R Clade D (*Vmn2r56*) | 5 | 3 | 88 | 4.5% | 4.2 ± 2.0 % |

**Table S2. Quantification of co-labeling in double *in situ* hybridization experiments with cell type-specific molecular markers.**
